# Supplementary material for: Meta-analysis towards FSHD reveals misregulation of neuromuscular junction, nuclear envelope, and spliceosome
Source: Commun Biol. 2024 May 25;7:640. doi: 10.1038/s42003-024-06325-z (PMC11127974; doi:10.1038/s42003-024-06325-z)
Supplement: Supplementary file 2 — Supplementary Information [file 42003_2024_6325_MOESM2_ESM.pdf]

Supplementary Notes

Meta-analysis towards FSHD reveals misregulation of neuromuscular junction, nuclear envelope, and spliceosome

Teresa Schätzl[1], Vanessa Todorow[2], Lars Kaiser[1], Helga Weinschrott[1], Benedikt Schoser[2], Hans-Peter Deigner[1,3,4], Peter Meinke[2], Matthias Kohl[1]

[1] Institute of Precision Medicine, Furtwangen University, Germany
[2] Friedrich-Baur-Institute at the Department of Neurology, LMU University Hospital, Ludwig Maximilian University, Munich, Germany
[3] Faculty of Science, Eberhard-Karls-University Tuebingen, Auf Der Morgenstelle 8, 72076, Tübingen, Germany
[4] EXIM Department, Fraunhofer Institute IZI (Leipzig), Schillingallee 68, 18057, Rostock, Germany

Table of contents

1. Meta-Analysis Materials & Methods 2
2. Quality Assessment Results 11
3. Investigation of Heterogeneity 15
4. Sensitivity Analyses 17
5. The Search for *DUX4* and *DUX4* Biomarker Genes in the *Meta-FSHD* App 19
6. Summary of the *Vote-Counting* Approach 21
7. Analysis of Results with and without Filter....................22

Supplementary Notes 1: Meta-Analysis Materials & Methods

Step 1: Literature Search

The meta-analysis follows the Preferred Reporting Items for Systematic Reviews and Meta-Analyses (PRISMA) Statement[1] and was registered in the international prospective register of systematic reviews (PROSPERO; ID: CRD42022330489). By May 2022, a comprehensive search for FSHD patient records was performed (four-eyes principle) in Gene Expression Omnibus (GEO)[2], ArrayExpress[3] and the European Genome-Phenome Archive (EGA)[4]. Duplicate publications were identified by comparing author names, titles, and publication dates. All studies were subsequently reviewed in detail for pre-defined parameters, which are described in section *2. Inclusion and Exclusion Criteria*. To classify the sequencing data, we emphasized descriptions of patients with regard to age, sex, D4Z4 repeats, pathogenic features or MRI data in case of respective MRI studies (see *Patient Information*, in section *2. Inclusion and Exclusion Criteria*). When patient tissue information was not available on the platforms, relevant publications based on the data were searched in databases such as PubMed, Medline, CINAHL, and EMBASE, as well as other relevant sources such as the Google search engine, Google Scholar, and World Health Organization websites, and were examined for additional background information on the samples. Furthermore, a total of five authors were contacted regarding inclusion or exclusion from the study due to missing patient information, of which two authors responded. One author provided the required data, whereas the other author was unable to provide additional study information. Furthermore, one study met the criteria but was not freely accessible in the EGA (EGAD00001008337[5]). After email request, we were informed that the corresponding *fastq* raw files could not be shared for privacy reasons, but we were kindly provided with the expression table and the metadata of the study (see section *4. Pre-Processing and Statistical Analysis*).

Step 2: Inclusion and Exclusion Criteria

General Data Frame. Datasets had to be of human origin, contain appropriate controls, and focus on FSHD1 (see **Figure 1**). Because of the slightly different pathological background of the FSHD1 and FSHD2 disease types, datasets containing solely FSHD2 samples were removed. However, individual FSHD2 samples were retained since *DUX4* expression has been described as a major feature of muscle wasting in both disease types [6-8] and some FSHD2 samples of the included studies show strong *DUX4* expression. So, by removing these samples, information could be lost (see *Supplementary Notes 2*). In this context, since only a few datasets (see *Supplementary Table 1*) include FSHD2 samples, retention of some FSHD2 samples may only account for general and not for disease-type-specific effects. This is supported by the *vote-counting* approach (see section *5. Data Synthesis*) as the results of our meta-analysis are based on a high rate of overlap of genes and molecular signalling pathways between the datasets.

Data from mosaic patients and embryonic stage data were excluded and the type of experiments was narrowed down to expression profiling by array or high-throughput sequencing.

Cell Stages. To date, it has been found that myoblasts and myotubes are affected very differently with respect to gene expression. Although FSHD myoblasts have been shown to be more sensitive to *DUX4* expression [9], recent evidence suggests that FSHD myotubes in particular have problems with oxidative phosphorylation in mitochondrial metabolism due to *DUX4* expression [10]. As the meta-analysis was intended to provide a comprehensive picture of skeletal muscle, biopsies or whole muscle models were primarily searched for, since they encompass all possible development stages of a muscle cell. However, the analysis was extended to cell line datasets when both myoblast and corresponding myotube data were available, since two main stages of myogenesis from satellite cell to fibre are represented in these[11-13]. This finally allowed comparison of the respective data with those of the biopsy datasets. In this context, we could not detect any differences between biopsy datasets and cell line datasets within the large amount of data. Nevertheless, it should be noted that the latter do not contain data on stem cells or specific interim forms of cell stages (see *Supplementary Notes 2*).

Patient Information. As FSHD is a rather moderately progressive disease compared with other muscular dystrophies and is characterized by unpredictable, individual-specific manifestations (e.g., involvement of specific muscles, asymmetries, severe or mild disease progression[14-16]), samples, which had no background information (neither on sex, age, D4Z4 repeats, pathological features nor on MRI data) were excluded. To refine the criteria for subsequent bioinformatic analysis strategies, datasets were divided into "*muscle biopsies*" and "*patient cell lines*" (primary/immortalized myoblasts and corresponding myotubes) depending on the cell material used and distinguished from "*DUX4 model systems*" since the latter do not depend on patient information due to model nature[17]. For the *DUX4* cell models, an inclusion criterion in the meta-analysis was that *DUX4* expression was examined in healthy muscle cells. Studies that analysed the effects of *DUX4* expression in cells marked by other diseases (like chronic myeloid leukaemia [18,19] or rhabdomyosarcoma [20]) were excluded. Ultimately, however, the *DUX4* models were not considered a true component of the meta-analysis (see *Supplementary Notes 2*), but were included for comparison with the patient datasets to draw possible conclusions about *DUX4*-dependent and *DUX4*-independent components.

Tissue. The involvement of specific muscle tissue has been shown to be highly variable in people with FSHD. This is also reflected in an often strong left-right asymmetry of individual muscle groups regarding the body axis [14-16]. Generally, FSHD initially affects the muscles of the face, shoulder girdle, and upper arms (mimetic muscles, serratus anterior and rhomboid muscles, and biceps and triceps). The disease is then described to progress over time and finally affect the lower extremities, typically the distal muscles

(tibialis anterior and gastrocnemius) first and later, the more proximal muscles (quadriceps and hamstrings) and the pelvic girdle. In contrast, the deltoid muscle is reported to be preserved or less affected [21,22]. Therefore, studies that solely focused on the deltoid muscle were excluded from our meta-analysis. Otherwise, no further restrictions were made since it became apparent within the research process that the remaining datasets examined muscle groups that are described as affected in FSHD depending on the time course.

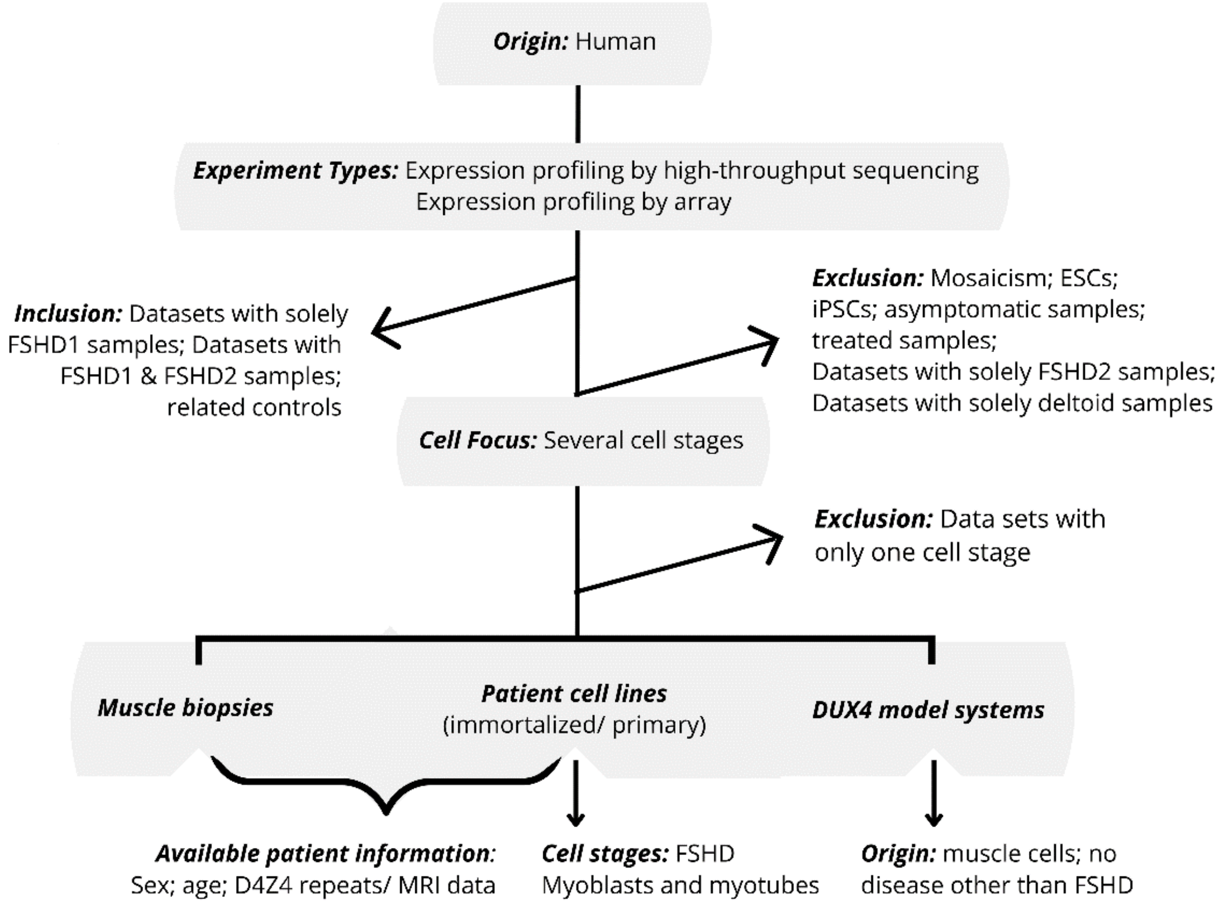

Figure 1. Inclusion and Exclusion Criteria. Cells had to be of human origin and the experiment type was narrowed down to "expression profiling by array or high-throughput sequencing"; Inclusion (on the left): Primarily FSHD1 patients with matched controls; Exclusion (on the right): Mosaicism, induced Pluripotent Stem Cells (iPSCs), Embryonic Stem Cells (ESCs), asymptomatic or treated samples and studies that examined solely deltoid samples or only one stage of cell development. Depending on the cells used, the categories "muscle biopsies", "patient cell lines" and "*DUX4* cell models" were formulated; All patient samples had to contain appropriate patient information and *DUX4* model systems had to be derived from healthy muscle tissue.

Step 3: Data Extraction and Quality Assessment

Titles and abstracts were screened by two independent reviewers (TS, HW; based on the four-eyes principle) who were carefully supervised by a third reviewer (MK). Full-text articles of relevant studies were independently assessed by two reviewers (TS, HW) before data were extracted from included studies.

In case of disagreement, a third reviewer (MK) was consulted until consensus was reached. An extraction form was used to collect relevant data, including publication information (dataset name, year of publication), technology, and number of samples plus controls. With regard to sample characteristics, ID, patient sex, patient age, D4Z4 repeats, muscle tissue and additional pathological features (if available) were collected (see *Supplementary Table 1*; results regarding an adjusted (adj.) p-value < 0.05[23,24] were recorded after data extraction within statistical analysis). In summary, a total of 11 studies were included in the meta-analysis [5,25-34]. 13 datasets were generated from the 11 studies; during data extraction, one dataset (GSE 56787[32]) was already split as it included both cell lines and biopsies and later, within statistical analysis (see section *4. Pre-processing and Statistical Analysis*), we split another dataset, (GSE 123468[30]), that encompassed two families, resulting in family-related batch effects due to strong genetic similarity in the families. The purpose of this meta-analysis was to make previous datasets comparable in terms of molecular effects and signalling pathways triggered within pathogenic expression in FSHD. The effect measurement was based on the relative risk in the form of the standardized log2-fold-change[35].

For quality analysis and the assessment of risk of bias, we followed the Cochrane criteria regarding qualitative evidence[36]. Since the original omics data were used for this meta-analysis, formerly different approaches to data analysis or outcome selection do not provide a target for potential bias. However, in addition to "*Bias in measurement of the outcome*" and "*Bias in the selection of the reported outcome*", four additional items were used for quality assessment: "*Bias due to missing outcome data*" when compromises had to be made regarding omics data originality, "*Bias due to different experimental designs*", when there were differences regarding the organisation of sequencing per se or involved cell tissues (biopsies *versus* cell lines; see *Cell Stages,* in section *2. Inclusion and Exclusion Criteria*) and "*Bias due to selection of participants*" when datasets included FSHD1 and FSHD2 samples (see *General Data Frame,* in section *2. Inclusion and Exclusion Criteria*). Finally, the item "*Bias due to different technology*" was included as the meta-analysis encompasses both different microarray technologies (Illumina and Affymetrix microarrays) as well as Illumina RNA-Seq technologies (see *Supplementary Notes 2*).

Step 4: Pre-Processing and Statistical Analysis

A uniform analysis procedure was performed, with pre-processing and analysis steps differing only in terms of the technology behind the datasets (Affymetrix GeneChips[37], Illumina BeadArray technology[38] or Illumina RNA-Seq[39]). All downstream analyses were performed with R (v4.2.2)[40]. The codes for pre-processing pipeline and reproducible analysis can be found in GitHub (https://github.com/FSHDresearch/Meta-Analysis-of-FSHD). For statistical analysis, the Benjamini-

Hochberg correction was used to adjust for multiple testing[24] and adj. p-values of < 0.05 were considered significant[23]. All statistical tests were two-sided.

In case of RNA-Seq data, Salmon (v1.9.0) was used to quantify transcript abundance from *fastq* raw files (which were freely available in the databases except for EGAD00001008337[5]), since it has been shown to significantly improve the accuracy of abundance estimates and the sensitivity of subsequent differential expression analysis[41]. In case of the EGA dataset, after email request we were provided with the raw counts table and the metadata (see section *1. Literature search*). The generation of raw values in this context had been described as follows: *"Reads were trimmed and quality filtered by TrimGalore (v0.4.5, cutadapt v1.16) using default parameters and mapped to Genome Reference Consortium Human Build 38 (GRCh38, Gencode release 28) using STAR aligner (v2.5.1b). A gene expression counts table was generated using HTSeq (v0.9.1, genome annotation hg38)."*[42] We integrated these raw data into the unified analysis procedure of our meta-analysis. In order not to lose data, no separate filtering steps were applied in the R workflows for the individual datasets[43]. For pre-processing Affymetrix microarray data, we used the Robust Multi-Array Average (RMA) approach[44] of the Bioconductor packages oligo (v1.60.0)[45] and affy (v1.74.0)[46]; and for Illumina microarray data we selected Robust Spline Normalization (RSN)[47] of the lumi package (v2.48.0)[48-51]. As we used limma (v3.52.2)[52] in combination with MKomics (v0.7)[53] for differential expression analysis of microarray data[54], we chose limma for RNA-Seq data preparation and analysis as well, since it required only minimal pipeline changes to switch between analyses for RNA-Seq and microarray experiments[55,56]. Limma is transforming the read counts to the logarithmic scale and empirically estimating the relationship between mean and variance. The mean-variance trend is transformed into precision weights by the voom function[57], which is integrated into the analysis of log-transformed RNA-Seq counts using the same linear modelling commands as for microarrays. Thus, this approach provides a basis for comparability between the different technologies, since the same statistical tests are available for both types of data, with the same format of results and graphical representations[54].

Step 5: Data Synthesis

For data synthesis all workflows can be found in GitHub (https://github.com/FSHDresearch/Meta-Analysis-of-FSHD). For data alignment, the Ensembl database version 108 (December 2022) was used.

Strategy 1. Random-Effects-Model. Due to a high technological heterogeneity (microarrays versus RNA-Seq technology) and the observed biological heterogeneity in FSHD[14-16,42], a *random-effects* model was selected to identify significantly differentially expressed genes between patients and controls. In this context, we used SMDH (*"standardized mean difference with heteroscedastic population variances in the two groups"*) as an effect measure, as suggested by Bonett (2009)[58]. We also examined the impact that a *fixed-effects* model would have on our meta-analysis (see *Supplementary Notes 3*)[59] and we performed sensi-

tivity analyses in order to validate the results (see *Supplementary Notes 4*). In summary, a total of 53113 unique Ensembl IDs could be identified. We decided to use only unique IDs that were measured in at least three datasets to get reliable results from the meta-analyses, which gave us 26858 unique IDs. We filtered our data to increase the power of our analysis [43,60] and included only those that showed at least a small effect, i.e., a standardized log2-FC (Cohen's d) of greater than 0.2[61], whereupon we obtained

13274 unique IDs. The use of the *random-effects* model led to 1935 significant results (adj. p-values < 0.05) when considering mean, standard deviation (SD) and sample size per dataset and group. These results are decreasingly sorted by SMDH (see *Supplementary Data 2*). In the further course, we used the Bioconductor *simplifyEnrichment* package to cluster and visualize the functional enrichment results [62]. Besides, corresponding enrichment analyses were performed using Gene Ontology (GO)-database [63,64] to find out significantly enriched molecular pathways in FSHD.

Strategy 2. Vote-Counting Approach. To validate the results of the *random-effects* model, a second alternative analysis approach, known as *"vote-counting"*[65], was performed. Here, the datasets were considered separately and the significant genes and molecular pathways were compared in terms of their overlap rate between datasets. 7 of the 13 datasets yielded significant results when considering the adj. p-values by using limma (v3.52.2)[52] (see *Supplementary Data 4*). In this context, standard annotation packages from Bioconductor were utilized to map the data to the ENSEMBLE gene ID[66]. The core strategy was to find out, which genes are altered in FSHD. Enrichment analyses were performed using GO-database [63,64] to identify molecular signalling pathways (see *Supplementary Data 1, sheets 5* and *6*).

References

1. Moher, D. *et al.* Preferred reporting items for systematic review and meta-analysis protocols (PRISMA-P) 2015 statement. *Revista Espanola de Nutricion Humana y Dietetica* **20**, 148–160 (2016).
2. Edgar, R., Domrachev, M. & Lash, A. E. *Gene Expression Omnibus: NCBI gene expression and hybridization array data repository*. vol. 30 http://www.ninds.nih.gov/ (2002).
3. Athar, A. *et al.* ArrayExpress update - From bulk to single-cell expression data. *Nucleic Acids Res* **47**, D711–D715 (2019).
4. Freeberg, M. A. *et al.* The European Genome-phenome Archive in 2021. *Nucleic Acids Res* **50**, D980–D987 (2022).
5. FSHD-group Leiden University Medical Center (LUMC). Dataset. RNA-sequencing data from human FSHD and control skeletal muscle biopsies. https://ega-archive.org/datasets/EGAD00001008337 (2022).
6. de Greef, J. C. *et al.* Clinical features of facioscapulohumeral muscular dystrophy 2. *Neurology* **75**, 1548 (2010).
7. Tawil, R. Chapter 97 - Facioscapulohumeral Dystrophy. in *Rosenberg's Molecular and Genetic Basis of Neurological and Psychiatric Disease (Fifth Edition)* (eds. Rosenberg, R. N. & Pascual, J. M.) 1169–1176 (Academic Press, 2015). doi:https://doi.org/10.1016/B978-0-12-410529-4.00097-8.

8. Yao, Z. *et al.* DUX4-induced gene expression is the major molecular signature in FSHD skeletal muscle. *Hum Mol Genet* **23**, 5342–5352 (2014).

9. Winokur, S. T. *et al.* Facioscapulohumeral muscular dystrophy (FSHD) myoblasts demonstrate increased susceptibility to oxidative stress. *Neuromuscular Disorders* **13**, 322–333 (2003).

10. Heher, P. *et al.* Interplay between mitochondrial reactive oxygen species, oxidative stress and hypoxic adaptation in facioscapulohumeral muscular dystrophy: Metabolic stress as potential therapeutic target. *Redox Biol* **51**, (2022).

11. Shahini, A. *et al.* Efficient and high yield isolation of myoblasts from skeletal muscle. *Stem Cell Res* **30**, 122–129 (2018).

12. Enwere, E. K., LaCasse, E. C., Adam, N. J. & Korneluk, R. G. Role of the TWEAK-Fn14-cIAP1-NF-κB signaling axis in the regulation of myogenesis and muscle homeostasis. *Front Immunol* **5**, (2014).

13. Jang, Y.-N. & Baik, E. J. JAK-STAT pathway and myogenic differentiation. *JAKSTAT* **2**, e23282 (2013).

14. Padberg, G. W., Lunt, P. W., Koch, M. C. & Fardeau, M. G. J. Diagnostic criteria for facioscapulohumeral muscular dystrophy. *Neuromuscular Disorders* **1**, 231–234 (1991).

15. Tawil, R. & Van Der Maarel, S. M. Facioscapulohumeral muscular dystrophy. *Muscle Nerve* **34**, 1–15 (2006).

16. Ricci, G. *et al.* Large scale genotype-phenotype analyses indicate that novel prognostic tools are required for families with facioscapulohumeral muscular dystrophy. *Brain* **136**, 3408–3417 (2013).

17. Jagannathan, S. *et al.* Model systems of DUX4 expression recapitulate the transcriptional profile of FSHD cells. *Hum Mol Genet* **25**, 4419–4431 (2016).

18. Ashoti, A., Geijsen, N., Vivié, J., Creyghton, M. & Baak, M. Series GSE154649. Next generation sequencing to reveal the transcriptome of a DUX4 inducible FSHD model. https://www.ncbi.nlm.nih.gov/geo/query/acc.cgi?acc=GSE154649 (2020).

19. Ashoti, A. *et al.* Series GSE155034. A genome-wide CRISPR/Cas phenotypic screen for modulators of DUX4 cytotoxicity reveals screen complications. https://www.ncbi.nlm.nih.gov/geo/query/acc.cgi?acc=GSE155034 (2020).

20. Shadle, S. *et al.* Series GSE87495. DUX4-induced dsRNA and MYC mRNA Stabilization Activate Apoptotic Pathways in Human Cell Models of Facioscapulohumeral Dystrophy. https://www.ncbi.nlm.nih.gov/geo/query/acc.cgi?acc=GSE87495 (2016).

21. Statland, J. M. & Tawil, R. Facioscapulohumeral Muscular Dystrophy. *Continuum (Minneap Minn)* **22**, 1916–1931 (2016).

22. Rahimov, F. *et al.* Transcriptional profiling in facioscapulohumeral muscular dystrophy to identify candidate biomarkers. *Proc Natl Acad Sci U S A* **109**, 16234–16239 (2012).

23. Krzywinski, M. & Altman, N. Points of significance: Significance, P values and t-tests. *Nat Methods* **10**, 1041–1042 (2013).

24. Benjamini, Y. & Hochberg, Y. Controlling the false discovery rate: a practical and powerful approach to multiple testing. *Journal of the royal statistical society series b-methodological* **57**, 289–300 (1995).

25. Watt, K. *et al.* Series GSE138768. DUX4 promotes mitochondrial impairment in skeletal muscle. https://www.ncbi.nlm.nih.gov/geo/query/acc.cgi?acc=GSE138768 (2021).

26. Ehrlich, M. & Tsumagari, K. Series GSE26145. Expression profiling FSHD vs. control myoblasts and myotubes. https://www.ncbi.nlm.nih.gov/geo/query/acc.cgi?acc=GSE26145 (2011).

27. Kho, A., Arashiro, P., Kunkel, L. & Zatz, M. Series GSE15090. Gene expression profiles in muscle tissue from FSHD patients. https://www.ncbi.nlm.nih.gov/geo/query/acc.cgi?acc=GSE15090 (2009).

28. Rahimov, F. Series GSE36398. Transcriptional profiling in facioscapulohumeral muscular dystrophy to identify candidate biomarkers. *2012* https://www.ncbi.nlm.nih.gov/geo/query/acc.cgi?acc=GSE36398.

29. Tasca, G., Pescatori, M., Cubeddu, T. & Ricci, E. Series GSE26852. Gene expression analysis of FSHD muscle with different MRI pattern. https://www.ncbi.nlm.nih.gov/geo/query/acc.cgi?acc=GSE26852 (2012).

30. Banerji, C. & Zammit, P. Series GSE123468. RNA-seq of FSHD and control immortalised myoblasts II. https://www.ncbi.nlm.nih.gov/geo/query/acc.cgi?acc=GSE123468 (2018).

31. Welle, S. Series GSE10760. Effect of facioscapulohumeral dystrophy (FSHD) on skeletal muscle gene expression. https://www.ncbi.nlm.nih.gov/geo/query/acc.cgi?acc=GSE10760 (2008).

32. Yao, Z. *et al.* Series GSE56787. DUX4-induced gene expression is the major molecular signature in FSHD skeletal muscle. https://www.ncbi.nlm.nih.gov/geo/query/acc.cgi?acc=GSE56787 (2014).

33. Cheli, S. & Meneveri, R. Series GSE26061. Expression profiling of 4q-linked and phenotypic FSHD in different steps of myogenic differentiation. https://www.ncbi.nlm.nih.gov/geo/query/acc.cgi?acc=GSE26061 (2011).

34. Freidman, S. *et al.* Series GSE115650. MRI-informed muscle biopsies correlate MRI with pathology and DUX4 target gene expression in FSHD. https://www.ncbi.nlm.nih.gov/geo/query/acc.cgi?acc=GSE115650 (2018).

35. Robinson, M. D. & Oshlack, A. *A scaling normalization method for differential expression analysis of RNA-seq data*. http://genomebiology.com/2010/11/3/R25 (2010).

36. The Cochrane Collaboration. Cochrane Training. Chapter 21: Qualitative evidence. https://training.cochrane.org/handbook/current/chapter-21 (2023).

37. Dalma-Weiszhausz, D. D., Warrington, J., Tanimoto, E. Y. & Miyada, C. G. DNA Microarrays, Part A: Array Platforms and Wet-Bench Protocols. [1] The Affymetrix GeneChip® Platform: An Overview. in *Methods in Enzymology* vol. 410 3–28 (Academic Press, 2006).

38. Fan, J. *et al.* DNA Microarrays, Part A: Array Platforms and Wet-Bench Protocols. [3] Illumina Universal Bead Arrays. in *Methods in Enzymology* vol. 410 57–73 (Academic Press, 2006).

39. Dillies, M.-A. *et al.* A comprehensive evaluation of normalization methods for Illumina high-throughput RNA sequencing data analysis. *Brief Bioinform* **14**, 671–683 (2013).

40. R Core Team. R: A language and environment for statistical computing. Available from: https://www.R-project.org/ (2022).

41. Patro, R., Duggal, G., Love, M. I., Irizarry, R. A. & Kingsford, C. Salmon provides fast and bias-aware quantification of transcript expression. *Nat Methods* **14**, 417–419 (2017).

42. van den Heuvel, A. *et al.* Facioscapulohumeral dystrophy transcriptome signatures correlate with different stages of disease and are marked by different MRI biomarkers. *Sci Rep* **12**, (2022).

43. Bourgon, R., Gentleman, R. & Huber, W. Independent filtering increases detection power for high-throughput experiments. *PNAS* **107**, (2010).

44. Irizarry, R. A. *et al. Exploration, normalization, and summaries of high density oligonucleotide array probe level data*. *Biostatistics* vol. 4 http://www.biostat.jhsph.edu/ (2003).

45. Carvalho, B. S. & Irizarry, R. A. A framework for oligonucleotide microarray preprocessing. *Bioinformatics* **26**, 2363–2367 (2010).

46. Gautier, L., Cope, L., Bolstad, B. M. & Irizarry, R. A. affy—analysis of Affymetrix GeneChip data at the probe level. *Bioinformatics* **20**, 307–315 (2004).

47. Du, P. & Lin, S. lumi: BeadArray Specific Methods for Illumina Methylation and Expression Microarrays. rsn: Robust Spline Normalization between chips. https://rdrr.io/bioc/lumi/man/rsn.html (2020).

48. Du, P., Kibbe, W. & Lin, S. lumi: a pipeline for processing Illumina microarray. *Bioinformatics* **24**, 1547–1548 (2008).

49. Du, P. *et al.* Comparison of Beta-value and M-value methods for quantifying methylation levels by microarray analysis. *BMC Bioinformatics* **11**, (2010).

50. Lin, S. M., Du, P., Huber, W. & Kibbe, W. A. Model-based variance-stabilizing transformation for Illumina microarray data. *Nucleic Acids Res* **36**, (2008).

51. Du, P., Kibbe, W. A. & Lin, S. M. NuID: A universal naming scheme of oligonucleotides for Illumina, Affymetrix, and other microarrays. *Biol Direct* **2**, (2007).

52. Smyth, G. K. *et al. limma: Linear Models for Microarray and RNA-Seq Data User's Guide*. (2021).

53. Kohl, M. MKomics: Omics Data Analysis. https://CRAN.R-project.org/package=MKomics (2021).

54. Ritchie, M. E. *et al.* Limma powers differential expression analyses for RNA-sequencing and microarray studies. *Nucleic Acids Res* **43**, e47 (2015).

55. Tong, Y. The comparison of limma and DESeq2 in gene analysis. *E3S Web of Conferences* **271**, 03058 (2021).

56. Corchete, L. A. *et al.* Systematic comparison and assessment of RNA-seq procedures for gene expression quantitative analysis. *Sci Rep* **10**, (2020).

57. Law, C. W., Chen, Y., Shi, W. & Smyth, G. K. *voom: precision weights unlock linear model analysis tools for RNA-seq read counts*. *Genome Biology* vol. 15 http://genomebiology.com/2014/15/2/R29 (2014).

58. Bonett, D. G. Meta-analytic interval estimation for standardized and unstandardized mean differences. *Psychol Methods* **14**, 225–238 (2009).

59. Borenstein, M., Hedges, L. V., Higgins, J. P. T. & Rothstein, H. R. A basic introduction to fixed-effect and random-effects models for meta-analysis. *Res Synth Methods* **1**, 97–111 (2010).

60. Hackstadt, A. J. & Hess, A. M. Filtering for increased power for microarray data analysis. *BMC Bioinformatics* **10**, (2009).

61. Cohen, J. *Statistical Power Analysis for the Behavioral Sciences*. (Routledge, 1988). doi:10.4324/9780203771587.

62. Gu, Z. & Hübschmann, D. Simplify enrichment: A bioconductor package for clustering and visualizing functional enrichment results. *Genomics Proteomics Bioinformatics* (2022) doi:https://doi.org/10.1016/j.gpb.2022.04.008.

63. Carbon, S. *et al.* The Gene Ontology resource: Enriching a GOld mine. *Nucleic Acids Res* **49**, D325–D334 (2021).

64. Ashburner, M. *et al.* Gene ontology: Tool for the unification of biology. *Nat Genet* **25**, 25–29 (2000).

65. Ramasamy, A., Mondry, A., Holmes, C. C. & Altman, D. G. Key issues in conducting a meta-analysis of gene expression microarray datasets. *PLoS Med* **5**, 1320–1332 (2008).

66. Aken, B. L. *et al.* The Ensembl gene annotation system. *Database (Oxford)* **2016**, (2016).

Supplementary Notes 2: Quality Assessment Results

The methodological quality of the studies is visualized by robvis web application [1] and is illustrated in **Figure 2** *Traffic Light Plot* and **Figure 3** *Summary Plot*. All references in this *Supplementary Notes 2* are referring to *Supplementary Notes 1*. By using original omics data, the risk of (1) *bias in measurement of the outcome* and (2) *selection of the reported outcomes* could be minimized. However, other parameters were considered more critical by the authors (see section *3. Data Extraction and Quality Assessment* in *Supplementary Notes 1*).

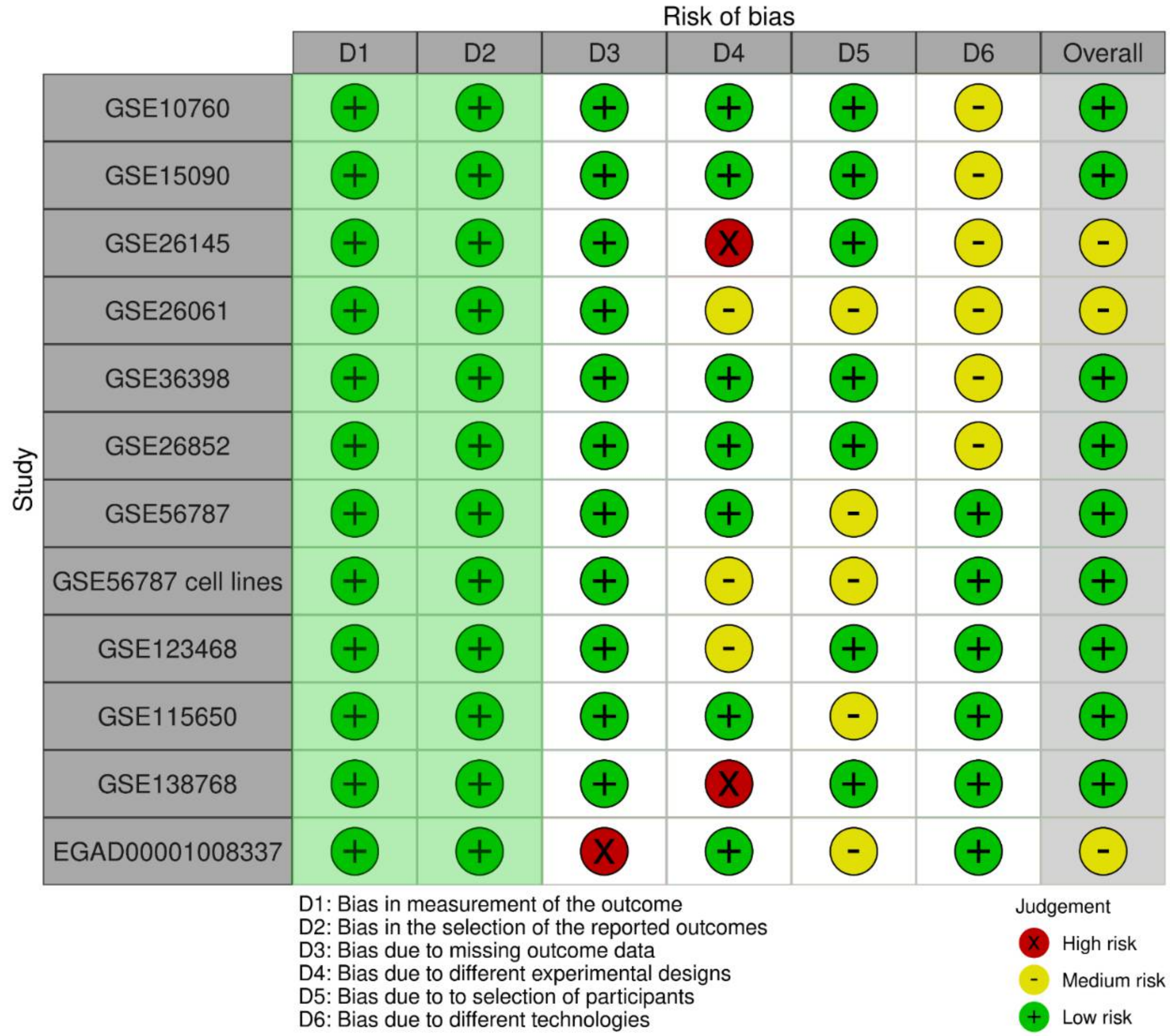

Figure 2. Quality Assessment by robvis: Traffic Light Plot. A total of 6 items were used to assess the quality of the study: (1) Measurement of outcomes, (2) selection of reported outcomes, (3) missing outcome data, (4) different experimental designs, (5) selection of participants and (6) different technologies. Each item was assessed as “low risk”, “unclear risk”, and “high risk”. Noticeable are datasets GSE26145 (high risk of bias due to different experimental design), GSE138768 (*DUX4* model system; high risk of bias due to model nature) and EGA00001008337 (high risk of bias due to missing outcome data).

Note: Of the 11 selected studies, one study was already split at the time of data preparation because it contained both cell lines and biospies. Another study was split later in the course of the statistical analysis, which is why 12 datasets (instead of the 13 datasets) are examined here.

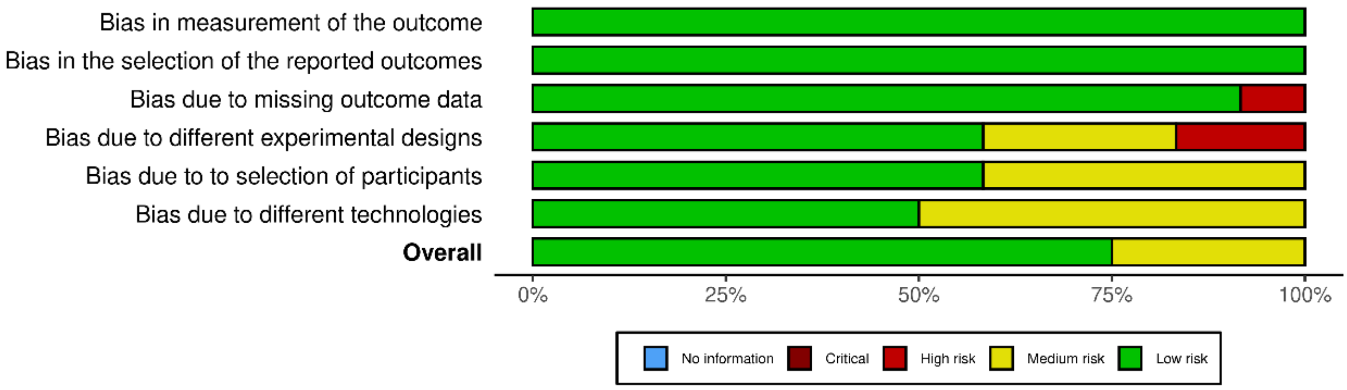

Figure 3. Quality Assessment by robvis: Summary Plot. While approximately 75% of the total data are assessed to be at low risk of potential bias, 25% are perceived to be at medium risk of bias; no studies are associated with high risk in the overall assessment.

Item 3: Bias due to missing outcome data

One study met the inclusion criteria for the meta-analysis but was not freely accessible in the EGA (EGAD00001008337[2]). After email request, we were informed that the corresponding *fastq* raw files could not be shared for privacy reasons, but we were kindly provided with the expression table and the metadata of the study (see section *4. Pre-Processing and Statistical Analysis*; in *Supplementary Notes 1*). In this case, data preparation and pre-processing differ from our approaches, potentially leading to bias.

Item 4: Bias due to different experimental designs

In terms of experimental design, some studies encompass biopsies (GSE10760, GSE15090, GSE36398, GSE26852, GSE56787_*biopsies*, GSE115650, EGAD00001008337) while others include cell lines (GSE26145, GSE26061, GSE56787_*cell lines*, GSE123468). Datasets on cell lines were included in the meta-analysis when both data on myoblasts and the data on myotubes developed from them, were available. Since two main stages of myogenesis from satellite cell to fibre are represented in these[3-5], this finally allowed comparison of the respective data with those of the biopsy datasets. However, in this context, there might be a risk of bias as the latter do not contain data on stem cells or specific interim forms of cell stages (see section *2. Inclusion and Exclusion Criteria*; in *Supplementary Notes 1*).

Besides, one dataset contains data from a *DUX4* model system, which has a high risk of bias due to its model nature (GSE138768[6]). Therefore, the *DUX4* model included in the meta-analysis was not included in the overall calculation, but was contrasted for comparison since *DUX4*-induced gene expression has been reported to be the major molecular signature in FSHD skeletal muscle[7].

Finally, regarding the sequencing process itself, one dataset is based on information of patients and controls, who were sequenced at two different time points (GSE26145[8]). Therefore, it could not be determined whether the resulting differences were batch effects due to the different dates of sequencing or actually

differences between patients and controls. For correcting data in this regard, we used ComBat of the Bioconductor package sva (v3.44.0)[9] having in mind that every batch correction may slightly change overall results.

Item 5: Bias due to selection of participants

In terms of patient selection, some studies included FSHD2 patients alongside FSHD1 patients, although the disease types are genetically slightly different (see section *2. Inclusion and Exclusion Criteria*; in *Supplementary Notes 1*). As some FSHD2 samples of the included studies show strong *DUX4* expression (see, e.g., *Supplemental Table 1* in Wang et al. (2019): *Subject Demographics, genetics, pathology and MRI features*[10]), by removing these samples, information could be lost. In this regard, in the *vote-counting* approach (see section *5. Data Synthesis*; in *Supplementary Notes 1*), the results of our meta-analysis are based on a high rate of overlap of genes and molecular signalling pathways between the datasets. Since only 5 of 13 datasets (see *Supplementary Table 1*) include FSHD 2 samples, retention of some FSHD 2 samples may only account for general and not for disease-type-specific effects. However, although the amount of FSHD2 samples is comparatively small (the 176 FSHD samples are composed of 142 FSHD1 samples and 25 FSHD2 samples), the differences have to be considered regarding potential future analysis based on our data.

Item 6: Bias due to different technologies

The meta-analysis encompasses Affymetrix microarrays (HG-U133A chip, HG-U133B chip, Human Genome U133 Plus 2.0 array, Human Exon 1.0 ST array, Human Gene 1.0 ST array), one Illumina microarray (HumanHT-12 v3 Expression BeadChip) and RNA-Seq technology (Illumina NextSeq500 and Illumina HiSeq 2500). In this context, studies have shown that RNA-Seq not only allows the study of novel transcripts, but also provides higher resolution, better coverage, and lower technical variability compared to microarrays[11-13]. The different technologies can therefore also represent a risk of bias.

In summary, precautions were taken with regard to the quality of the results (batch corrections, exclusion of datasets, see *Bias due to different experimental designs* or use of different analysis approaches, see *Bias due to selection of participants*). With regard to the further procedure in the statistical analysis, comparison parameters were used (*random-effects* model vs. *fixed-effects* model, see *Supplementary Notes 3*) and sensitivity analyses were performed (see *Supplementary Notes 4*) to support the quality of the results. In general, two different analysis methods, the *random-effects* model [14-16] and the *vote-counting* approach [17], were performed, to validate the results of the meta-analysis.

References

1. McGuinness, L. A. & Higgins, J. P. T. Risk-of-bias VISualization (robvis): An R package and Shiny web app for visualizing risk-of-bias assessments. *Res Synth Methods* **12**, 55–61 (2021).
2. FSHD-group Leiden University Medical Center (LUMC). Dataset. RNA-sequencing data from human FSHD and control skeletal muscle biopsies. https://ega-archive.org/datasets/EGAD00001008337 (2022).
3. Shahini, A. *et al.* Efficient and high yield isolation of myoblasts from skeletal muscle. *Stem Cell Res* **30**, 122–129 (2018).
4. Enwere, E. K., LaCasse, E. C., Adam, N. J. & Korneluk, R. G. Role of the TWEAK-Fn14-cIAP1-NF-κB signaling axis in the regulation of myogenesis and muscle homeostasis. *Front Immunol* **5**, (2014).
5. Jang, Y.-N. & Baik, E. J. JAK-STAT pathway and myogenic differentiation. *JAKSTAT* **2**, e23282 (2013).
6. Watt, K. *et al.* Series GSE138768. DUX4 promotes mitochondrial impairment in skeletal muscle. https://www.ncbi.nlm.nih.gov/geo/query/acc.cgi?acc=GSE138768 (2021).
7. Yao, Z. *et al.* DUX4-induced gene expression is the major molecular signature in FSHD skeletal muscle. *Hum Mol Genet* **23**, 5342–5352 (2014).
8. Ehrlich, M. & Tsumagari, K. Series GSE26145. Expression profiling FSHD vs. control myoblasts and myotubes. https://www.ncbi.nlm.nih.gov/geo/query/acc.cgi?acc=GSE26145 (2011).
9. Leek, J. T., Johnson, W. E., Parker, H. S., Jaffe, A. E. & Storey, J. D. The SVA package for removing batch effects and other unwanted variation in high-throughput experiments. *Bioinformatics* **28**, 882–883 (2012).
10. Wang, L. H. *et al.* MRI-informed muscle biopsies correlate MRI with pathology and DUX4 target gene expression in FSHD. *Hum Mol Genet* **28**, 476–486 (2019).
11. Corchete, L. A. *et al.* Systematic comparison and assessment of RNA-seq procedures for gene expression quantitative analysis. *Sci Rep* **10**, (2020).
12. Perkins, J. R. *et al.* A comparison of RNA-seq and exon arrays for whole genome transcription profiling of the L5 spinal nerve transection model of neuropathic pain in the rat. *Mol Pain* **10**, (2014).
13. Zhao, S., Fung-Leung, W. P., Bittner, A., Ngo, K. & Liu, X. Comparison of RNA-Seq and microarray in transcriptome profiling of activated T cells. *PLoS One* **9**, (2014).
14. Viechtbauer, W. The metafor Package. A Meta-Analysis Package for R. h https://metafor-project.org/doku.php/metafor. (2021).
15. Borenstein, M., Hedges, L. V., Higgins, J. P. T. & Rothstein, H. R. A basic introduction to fixed-effect and random-effects models for meta-analysis. *Res Synth Methods* **1**, 97–111 (2010).
16. Bonett, D. G. Meta-analytic interval estimation for standardized and unstandardized mean differences. *Psychol Methods* **14**, 225–238 (2009).
17. Ramasamy, A., Mondry, A., Holmes, C. C. & Altman, D. G. Key issues in conducting a meta-analysis of gene expression microarray datasets. *PLoS Med* **5**, 1320–1332 (2008).

Supplementary Notes 3: Investigation of Heterogeneity

In general, a total of 53113 unique Ensembl IDs could be identified (Ensembl database version 108 from December 2022). We decided to use only unique IDs measured in at least three datasets to obtain reliable results from the meta-analyses, which gave us 26858 unique IDs. We filtered our data to increase the power of our analysis [1,2] and included only those that showed at least a small effect, i.e., a standardized log2-FC (SMDH) of greater than 0.2[3]. In this way, 13274 unique IDs were obtained and could be considered in the analysis of the *random-effects* model. (As can be seen from the **Table 1**, there is overlap between the Cochrane heterogeneity classification categories, which is why adding up the individual categories results in a slightly larger number of genes).

Table 1. *Random-effects* model based meta-analysis genes in context of heterogeneity[4]

Cochrane Heterogeneity Classification		Meta-Analysis: number of genes	Percentage (%)
0% to 40%	might not be important	6447	50.1
30% to 60%	moderate heterogeneity	4055	30.5
50% to 90%	substantial heterogeneity	5270	39.7
75% to 100%	considerable heterogeneity	1457	11.0

In principle, of the 13274 genes for which the meta-analysis was performed, 6447 (50.1%) of the genes have heterogeneity of 0-40%, which might not be important according to Deeks et al. in chapter 10 *Analysing data and undertaking meta-analyses* of the Cochrane Handbook (version 6.3, 2022). As depicted in the **Table 1**, we went with a *random -effects* model instead of a *fixed -effects* model since the remaining portion of genes were found to be heterogeneous and differences in heterogeneity could be detected : In 4055 (30 .5%) of the genes , heterogeneity of 30 -60 % is present , which is referred to as moderate heterogeneity according to the Cochrane criteria; in 5270 (39.7%) of the genes, heterogeneity of 50-90% is present, which is referred to as substantial heterogeneity; and in 1457 (11.0%) of the genes there is 75- 100% heterogeneity, which is referred to as considerable heterogeneity.[4]

Using a *fixed-effects* model, there would be 8241 genes (out of the 13274) that would have a p-value < 0.05 and 7047 genes that would have an adjusted p-value < 0.05, making the model very liberal. The *random-effects* analysis is more conservative. All 1935 genes from the *random-effects* analysis are also significant in the *fixed-effects* analysis.[4,5]

References

1. Bourgon, R., Gentleman, R. & Huber, W. Independent filtering increases detection power for high-throughput experiments. *PNAS* **107**, (2010).
2. Hackstadt, A. J. & Hess, A. M. Filtering for increased power for microarray data analysis. *BMC Bioinformatics* **10**, (2009).

3. Bonett, D. G. Meta-analytic interval estimation for standardized and unstandardized mean differences. *Psychol Methods* **14**, 225–238 (2009).

4. Deeks, J., Higgins, J. & Altman, D. Chapter 10: Analysing data and undertaking meta-analyses. in *Cochrane Handbook for Systematic Reviews of Interventions* (eds. Higgins, J. et al.) vol. Version 6.3 (2022).

5. Borenstein, M., Hedges, L. V., Higgins, J. P. T. & Rothstein, H. R. A basic introduction to fixed-effect and random-effects models for meta-analysis. *Res Synth Methods* **1**, 97–111 (2010).

Supplementary Notes 4: Sensitivity Analyses

Table 2: Distribution of the 1935 significant results of the *random-effects* model. *(The DUX4 model is not included in the calculation and is not mentioned here.)*

Datasets	3	4	5	6	7	8	9	10	11	12
Genes	143	189	54	151	73	65	116	296	28	820

As the **Table 2** shows, the 1935 significant genes of the *random-effects* model (see *Supplementary Data 1, sheet 2*) are present in multiple datasets. There are genes that are only present in 3 datasets (143) and there are genes that are present in all datasets included in the meta-analysis (820). Overall, 80.1% of the significant genes are based on six or more datasets, 65.1% are based on nine or more datasets, and 42.4% of the significant genes are present in all 12 datasets.

First, we applied the regression test by Egger et al. (1997) to examine the asymmetry of funnel plots, which may be an indicator of publication bias. 1099 of the 13274 meta-analyses yielded a p-value < 0.05, but no significant result remained after adjusting the p-values by the false discovery rate (FDR). Furthermore, Egger's test was significant (raw p-value < 0.05) in only 121 (6.3%) of the 1935 meta-analyses with a significant effect. In addition, trim and fill analyses were performed using the L0 estimator introduced by Duval et al (2000)[1]. One or more studies were added in 46.8% (6206 of 13274) of the meta-analyses, with only one or two studies added in most cases (4817 of 6206). Because the trim-and-fill method tends to overestimate asymmetry[2], we interpreted this result as only a minor indication of the presence of publication bias. Finally, we used the LFK index[3], which revealed a minor asymmetry in 1483 (11.2%) of all and in 250 (12.9%) of the significant meta-analyses. Major asymmetry was found in 597 (4.5%) of all and in 86 (4.4%) of the significant meta-analyses. Overall, we mainly found evidence of no or minor asymmetry and therefore do not expect a relevant publication bias in our meta-analysis.

Furthermore, according to Ramasamy et al. (2008), it is recommended to perform leave-one-out (LOO) analysis to examine the sensitivity of results to individual studies[4]. Since we had split two datasets (GSE56787 and GSE123468; see *Supplementary Notes 1*), we performed the LOO analyses at both the dataset level (*12 datasets*) and the study level (*10 studies*).

LOO analyses at dataset level

In total, 1568 of the 1935 genes are significant (81.0%) in all LOO analyses. However, as the meta-analysis only considers genes if they are measured in at least 3 datasets, in fact, only the 1935 *minus* 143 genes can be used here, since the 143 genes from meta-analyses of only 3 datasets will not appear in the intersection

of all LOO analyses (87.5%). Thus, far more than 80% of the significant genes are also significant in all LOO analyses.

LOO analyses at study level

The 143 genes are also omitted from the study-level LOO analysis. Of the 1935 genes that are significant in all LOO analyses, there are 1553 genes, or 80.3%, (or 86.7% if the 143 genes are excluded). So again, more than 80% of the genes are significant, suggesting very robust results.

References

1. Duval, S. & Tweedie, R. A Nonparametric "Trim and Fill" Method of Accounting for Publication Bias in Meta-Analysis. *J Am Stat Assoc* **95**, 89–98 (2000).
2. Zwetsloot, P.-P. *et al.* Standardized mean differences cause funnel plot distortion in publication bias assessments. *Elife* **6**, e24260 (2017).
3. Furuya-Kanamori, L., Barendregt, J. J. & Doi, S. A. R. A new improved graphical and quantitative method for detecting bias in meta-analysis. *JBI Evid Implement* **16**, (2018).
4. Ramasamy, A., Mondry, A., Holmes, C. C. & Altman, D. G. Key issues in conducting a meta-analysis of gene expression microarray datasets. *PLoS Med* **5**, 1320–1332 (2008).

Supplementary Notes 5: The Search for *DUX4* and *DUX4* Biomarker Genes in the *Meta-FSHD* App

Although *DUX4* is a central aspect of the disease, detection is extremely difficult since it is expressed sporadically and only in a few myonuclei before initiating signaling cascades that unleash its devastating effects[1] (see chapter *Confirmation of previous knowledge of FSHD pathology* in the main text). Therefore, *DUX4* biomarker genes, such as those described in Yao et al. (2014)[2] or Wang et al. (2019)[3], must be used to study *DUX4* expression, also when using the *meta-FSHD* app. Another point to consider is the fact that some genes may only appear in RNA-Seq technologies but not in the microarrays since RNA-Seq generally has technical advantages in terms of resolution and coverage[4-6]. It also allows screening for novel transcripts and is not based on predefined gene libraries. This is of great importance since *DUX4* and its biomarker genes are active during embryogenesis but are mostly silenced during life, which is why they are either missing on the microarray chips or not comparable due to outdated annotations. However, this does not diminish the performance of the microarrays in terms of downstream effects, important genes or mechanisms in FSHD, as shown by our sensitivity analyses (see *Supplementary Notes 4*). Nevertheless, this could be the reason why *DUX 4* biomarker genes are highly overexpressed in the RNA-Seq datasets but absent in the microarrays. In this regard, the app also shows extreme numbers, which is due to the fact that the expression in controls is close to zero.

Since *DUX4* appears to be a central component in FSHD, we also included one *DUX4* model in our meta-analysis as it met our predefined criteria. However, to avoid biasing the results of the meta-analysis by the extreme influence of the *DUX4* model on *DUX4* expression, we did not include the *DUX4* model in the *random-effects* calculation. Nevertheless, as depicted in *Figure 1d* in the main text, it is listed separately in the app to provide the opportunity of directly comparing it to the patient datasets. This provides valuable insights because although *DUX4* appears to be a core component of FSHD pathology, there are a number of genes that express opposite to the artificially created model (see chapter *New insights into FSHD* in the main text), possibly indicating a role independent of DUX4 expression. Using the app, researchers can thus obtain information from both the *DUX4* model and the patient datasets, also in case of yet unknown secondary players, which may act independent from *DUX4*.

References

1. Tassin, A. *et al.* DUX4 expression in FSHD muscle cells: How could such a rare protein cause a myopathy? *J Cell Mol Med* **17**, 76–89 (2013).
2. Yao, Z. *et al.* Series GSE56787. DUX4-induced gene expression is the major molecular signature in FSHD skeletal muscle. https://www.ncbi.nlm.nih.gov/geo/query/acc.cgi?acc=GSE56787 (2014).
3. Wang, L. H. *et al.* MRI-informed muscle biopsies correlate MRI with pathology and DUX4 target gene expression in FSHD. *Hum Mol Genet* **28**, 476–486 (2019).

4. Corchete, L. A. *et al.* Systematic comparison and assessment of RNA-seq procedures for gene expression quantitative analysis. *Sci Rep* **10**, (2020).

5. Perkins, J. R. *et al.* A comparison of RNA-seq and exon arrays for whole genome transcription profiling of the L5 spinal nerve transection model of neuropathic pain in the rat. *Mol Pain* **10**, (2014).

6. Zhao, S., Fung-Leung, W. P., Bittner, A., Ngo, K. & Liu, X. Comparison of RNA-Seq and microarray in transcriptome profiling of activated T cells. *PLoS One* **9**, (2014).

Supplementary Notes 6: Summary of the *Vote-Counting* Approach

Since our meta-analysis provided several new insights into FSHD, we performed an alternative analysis approach known as *"vote-counting"*[1], to validate our results. In this case, datasets were considered individually to determine the rate of overlap of significantly differentially expressed genes and pathways between FSHD patients and controls. This allowed the individual technologies and patient information to be considered in an overall result per dataset. In this regard, some datasets within our statistical analysis, had no adj. p-values < 0.05 (see *Discussion* in the main text). However, 7 datasets yielded significant results (see *Supplementary Data 1, sheets 4, 5* and *6* based on results of workflows published in https://github.com/FSHDresearch/Meta-Analysis-of-FSHD) and surprisingly, all the results we had obtained with our *random-effects* model persisted, although we certainly lost power when looking at the comparatively small sample sizes of individual datasets compared to our *random-effects* model, which encompassed a total of 292 samples (plus 8 *DUX4* model samples).

In case of mitochondrial membrane potential regulation, there is a high rate of overlap (6 of 7 datasets) in the upregulated pathways (see *Supplementary Data 1, sheet* 5), whereas *various* terms involving metabolism are in the upper range of the downregulated pathways (see *Supplementary Data 1, sheet* 6). An imbalance at the NMJ is supported by genes like *AGRN*, *DVL1* or *MACF1* (in 5 of the 7 datasets; see *Supplementary Data 1, sheet* 4) and upregulated signalling pathways such as *"acetylcholine receptor signalling"* and *"cellular response to acetylcholine"* (in 5 of the 7 datasets; see *Supplementary Data 1, sheet* 5). In addition to terms such as e.g. *"nuclear body"* or *"nuclear speck"* (in 4 of 7 datasets) hinting on genome organization, there is a large overlap in down-regulated pathways within the single datasets associated with splicing events (see *Supplementary Data 1, sheet* 6). This is particularly interesting since the results of the *random-effects* analysis had also shown "spliceosomal complex" alongside "nuclear body" and "nuclear speck" among the top 25 downregulated CC clusters (see *Supplementary Data 1, sheet* 3).

Reference

1. Ramasamy, A., Mondry, A., Holmes, C. C. & Altman, D. G. Key issues in conducting a meta-analysis of gene expression microarray datasets. *PLoS Med* **5**, 1320–1332 (2008).

Supplementary Notes 7: Analysis of Results with and without Filter

A filter was used in the meta-analysis to increase the significance and obtain a more comprehensive picture of the biological processes[1,2]. It seemed difficult to use a signal or variance filter without potentially increasing technical bias as we would have to find independent, cross-technology cut-offs. Hence, a standardized fold-change filter (FC filter) was chosen with a technology independent cut-off of 0.2, which corresponds to a small effect size in terms of Cohen's d[3]. It is reported that FC filters increase power more than signal or variance filters. However, FC filters can change the distribution of the p-values under the null hypothesis. In this case, a bias must be expected when calculating the false discovery rate (FDR) with the usual methods [4]. We are not aware of any method with which the FDR can be calculated directly for the filtered dataset in this case.

Therefore, a comparison between the analyses with and without FC filter is presented here. The distributions of the p-values are depicted in **Figure 4.**

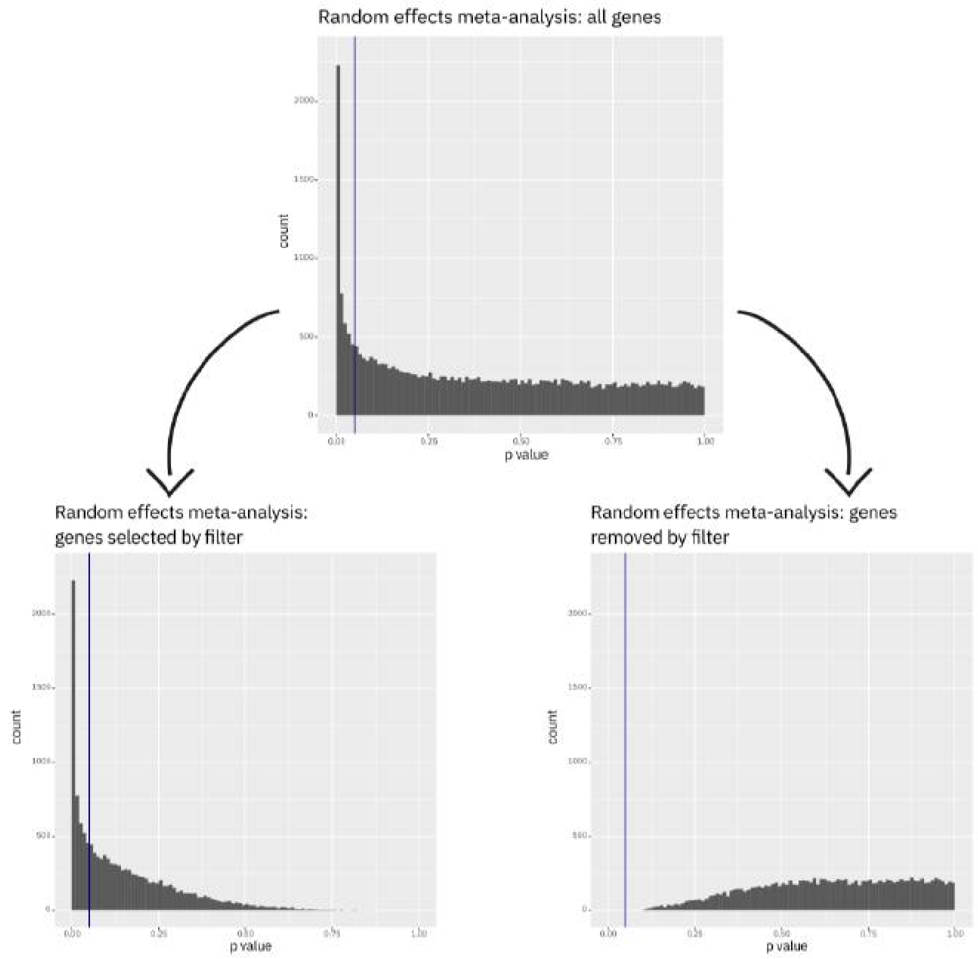

Figure 4. **Histograms of p-values.** The meta-analysis with random effects with all genes (top) is divided into "genes selected by filter" (bottom left) and "genes removed by filter" (bottom right). The blue line is located at p = 0.05.

The FC filter did not remove a single gene with a p-value < 0.05. The smallest p-value of the removed genes is 0.105. In other words, if we sort the p-values in ascending order and restrict ourselves to all genes with p-values < 0.05, this results in the same number of "significant" genes independent of filtering, namely 4571 genes. If we consider the 1935 genes that have an FDR < 0.05 after filtering

(calculated with Benjamini-Hochberg (BH)[5]), this would correspond to an FDR of 10.1% in the analysis without the filter (see *Supplementary Data 1, sheets 2a and 2b*). Since the FC filter reduces the overall proportion of null p-values (π_0)[4], we can expect that the actual FDR is less than 10.1%. Therefore , we tried to estimate the true FDR of our analysis with FC filter . For the estimation , we used the full dataset without filtering and applied different approaches: The method of Dalmasso et al. (2005) yields an FDR estimate of 6.8% (π_0 = 67.2%) and an upper bound of 8.0% (π_0 = 79.7%)[6,7] for the 1935 genes. The q-value approach of Storey (2002) results in estimated FDR of 7.3% (π_0 = 72.2%)[8,9,10]. The "last histogram height" approach of Murray and Blume (2021) gives 7.2% (π_0 = 71.4%)[11,12].

The above results for the unfiltered dataset suggest that the actual FDR for our 1935 genes is slightly higher than 5% and might be in the range of 7%. However, assuming that the FC filter predominately removed genes originating from the null hypothesis[4], the true proportion of null p-values π_0 in the filtered dataset can be expected to be smaller than estimated above. In summary, we therefore expect the true FDR of our analysis to be in the range of 5% to 7%.

References

1. Bourgon, R., Gentleman, R. & Huber, W. Independent filtering increases detection power for high-throughput experiments. *PNAS* **107**, 9546-51 (2010).

2. Hackstadt, A.J. & Hess, A.M. Filtering for increased power for microarray data analysis. *BMC Bioinformatics* **10**, 11 (2009).

3. Cohen, J. Statistical Power Analysis for the Behavioral Sciences. (Routledge, 1988).

4. van Iterson, M., Boer, J.M. & Menezes, R.X. Filtering, FDR and power. *BMC Bioinformatics* **11**, 450 (2010).

5. Benjamini, Y. & Hochberg, Y. Controlling the false discovery rate: a practical and powerful approach to multiple testing. *Journal of the royal statistical society series b-methodological* **57**, 289–300 (1995).

6. Dalmasso, C., Broët, P. & Moreau, T. A simple procedure for estimating the false discovery rate. *Bioinformatics (Oxford, England)* **21**, 660–668 (2005).

7. Dalmasso C. LBE: Estimation of the false discovery rate. R package version 1.68.0, https://bioconductor.org/packages/LBE (2023).

8. Storey J.D. A direct approach to false discovery rates. *Journal of the Royal Statistical Society, Series B* **64**, 479-498 (2002).

9. Storey J.D. and Tibshirani R. Statistical significance for genome-wide experiments. *Proceedings of the National Academy of Sciences* **100**, 9440-9445 (2003).

10. Storey J.D., Bass A.J., Dabney A., Robinson D. qvalue: Q-value estimation for false discovery rate control. R package version 2.32.0,https://bioconductor.org/packages/qvalue (2023).

11. Murray M.H. and Blume J.D. FDRestimation: Flexible False Discovery Rate Computation in R [version 2; peer review: 2 approved]. *F1000Research* **10**, 441 (2021).

12. Murray M., Blume J. FDRestimation: Estimate, Plot, and Summarize False Discovery Rates. R package version 1.0.1, https://CRAN.R-project.org/package=FDRestimation (2022).

Supplementary Figures

Meta-analysis towards FSHD reveals misregulation of neuromuscular junction, nuclear envelope, and spliceosome

Teresa Schätzl[1], Vanessa Todorow[2], Lars Kaiser[1], Helga Weinschrott[1], Benedikt Schoser[2], Hans-Peter Deigner[1,3,4], Peter Meinke[2], Matthias Kohl[1]

[1] Institute of Precision Medicine, Furtwangen University, Germany

[2] Friedrich-Baur-Institute at the Department of Neurology, LMU University Hospital, Ludwig Maximilian University, Munich, Germany

[3] Faculty of Science, Eberhard-Karls-University Tuebingen, Auf Der Morgenstelle 8, 72076, Tübingen, Germany

[4] EXIM Department, Fraunhofer Institute IZI (Leipzig), Schillingallee 68, 18057, Rostock, Germany

Table of contents

1. GO Biological Process (BP)..2

2. GO Cellular Component (CC) 4

3. GO Molecular Function (MF) 6

4. FSHD T2-STIR- Samples Expressing like Controls 8

5. GOChord Splicing FSHD_group3..9

6. GOChord Splicing FSHD_group2..10

Supplementary Figure 1a: GO **BP** - FSHD down

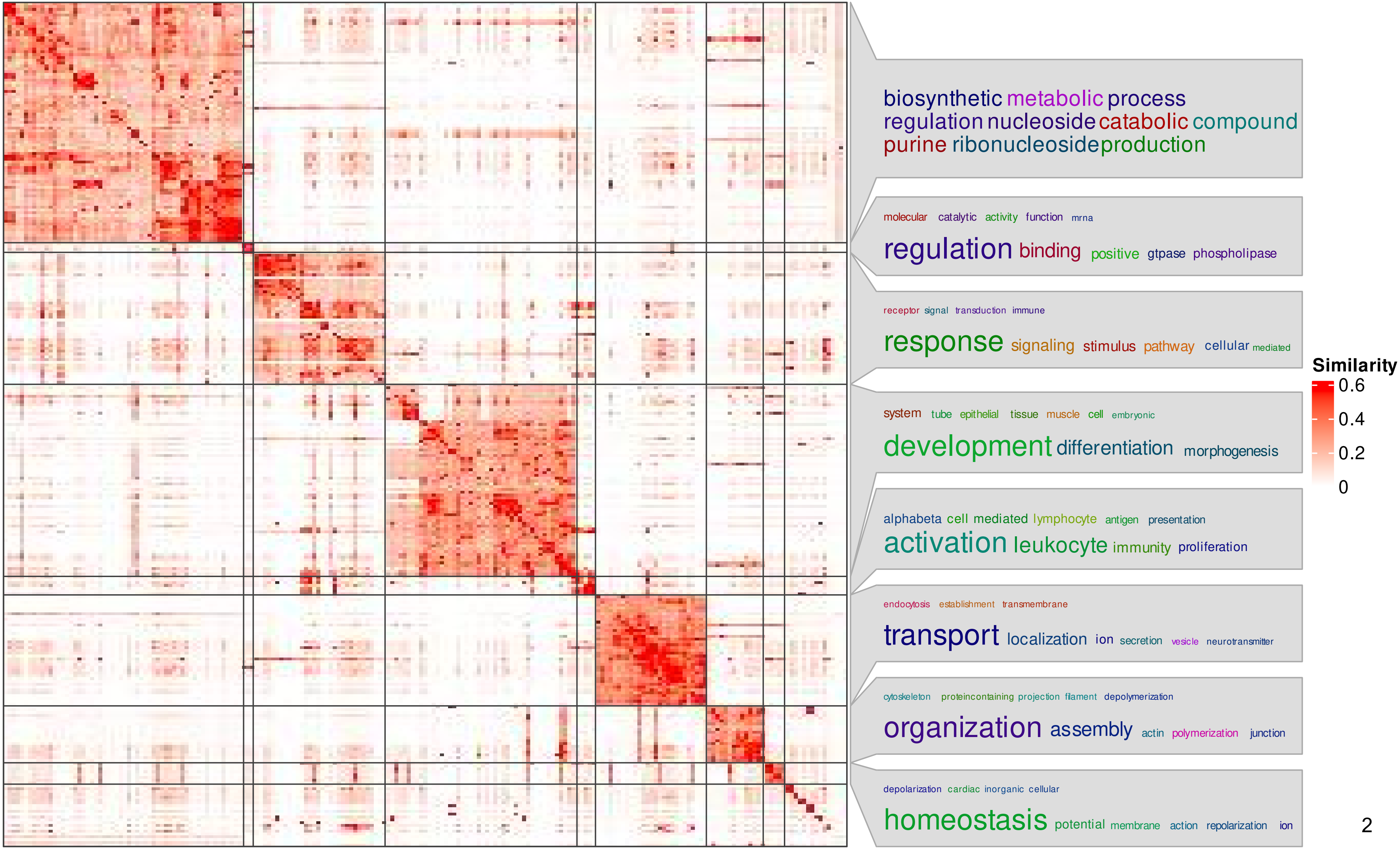

Supplementary Figure 1b: GO **BP** - FSHD up

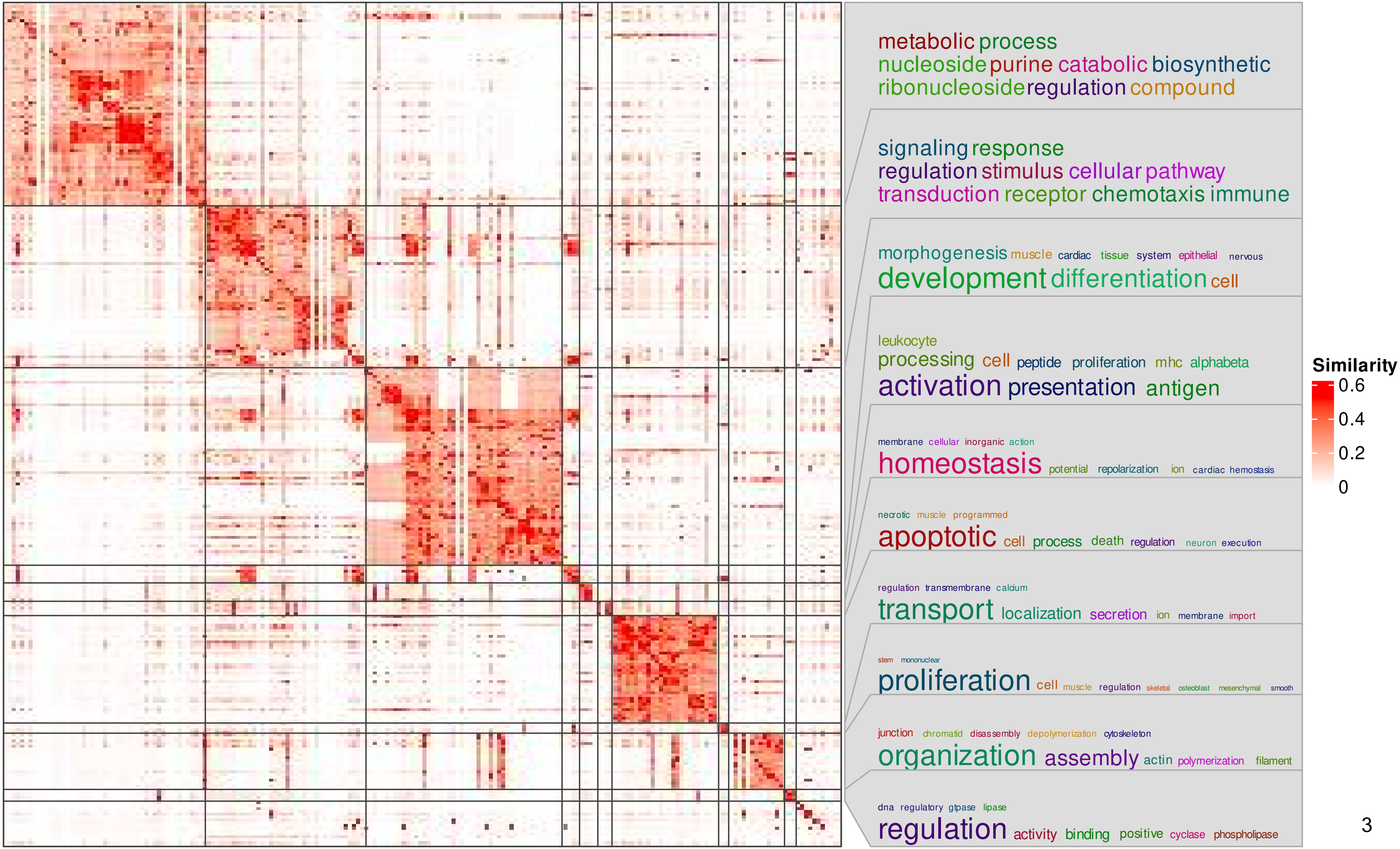

Supplementary Figure 2a: GO **CC** - FSHD down

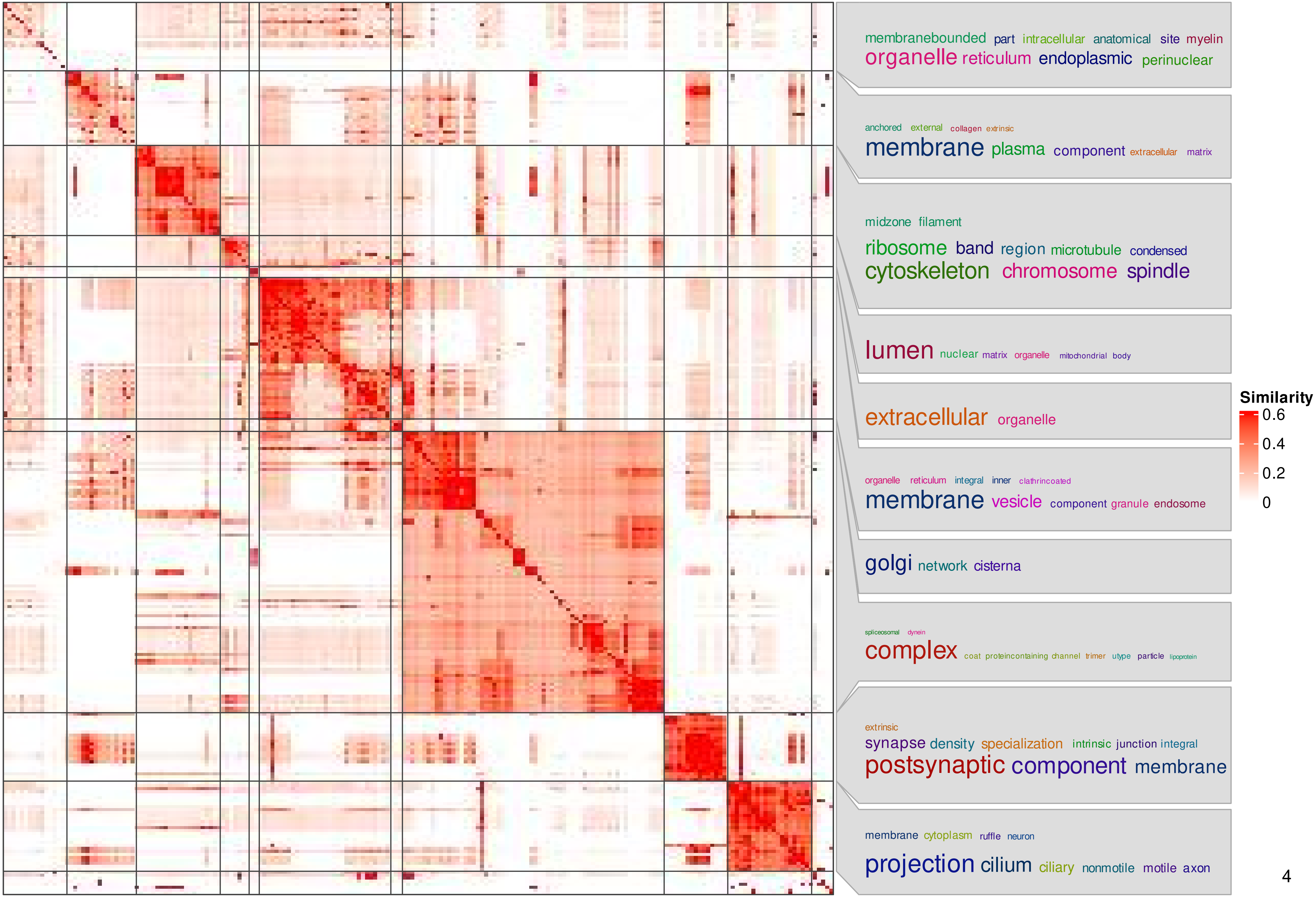

Supplementary Figure 2b: GO **CC** - FSHD up

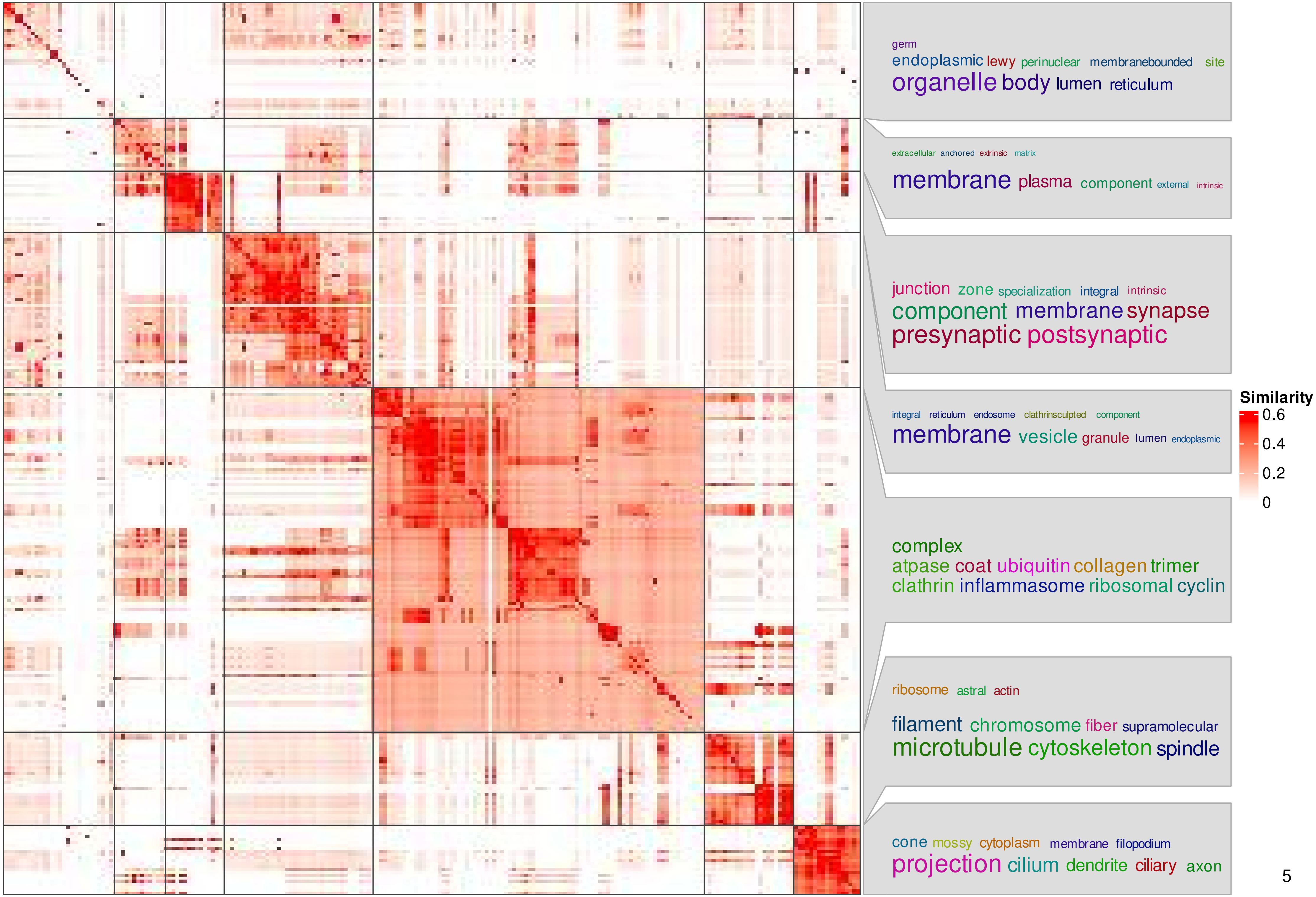

Supplementary Figure 3a: GO **MF** - FSHD down

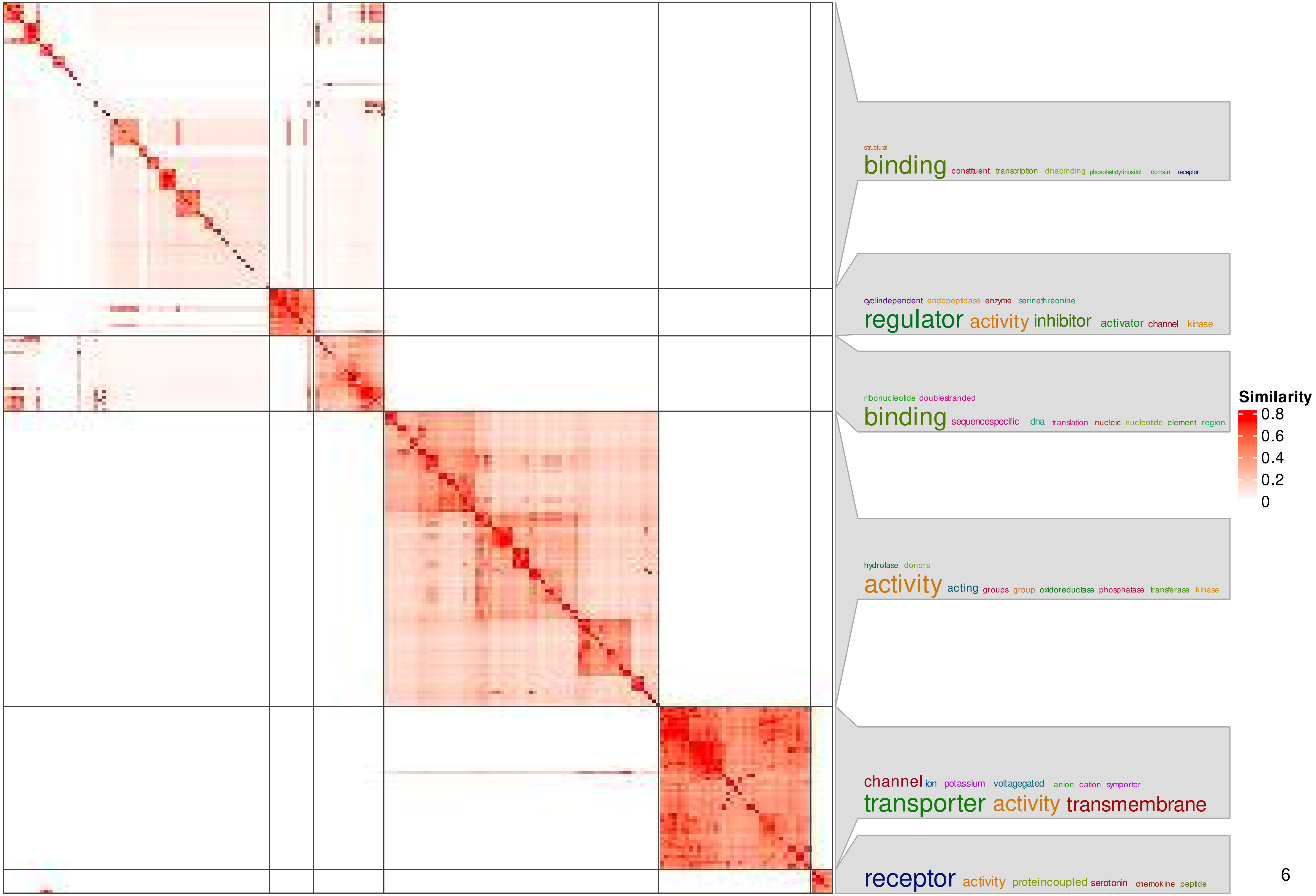

Supplementary Figure 3b: GO **MF** - FSHD up

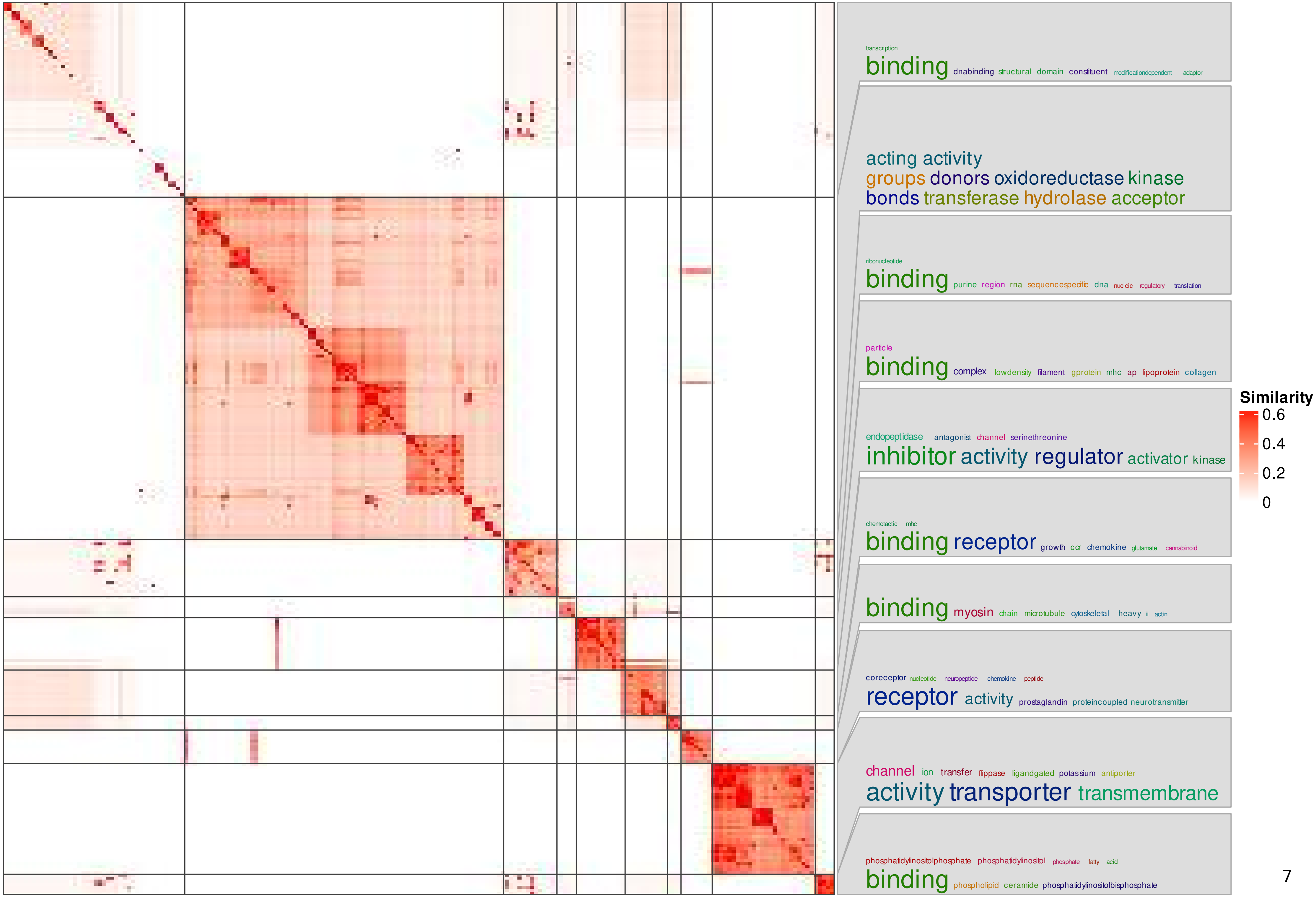

Supplementary Figure 4: FSHD T2-STIR- Samples Expressing like Controls

Adjusted p-value < 0.05

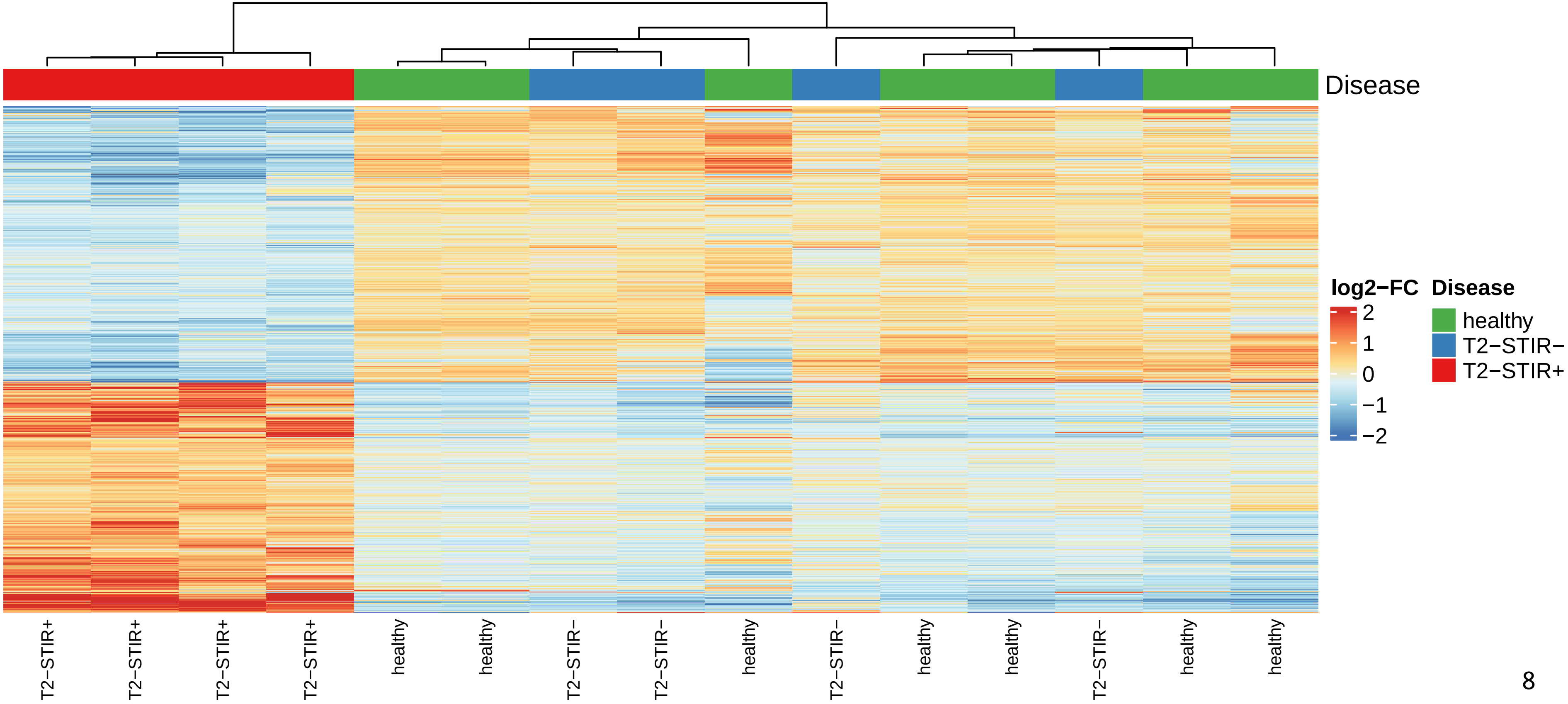

Supplementary Figure 5: GOChord Splicing FSHD_group3

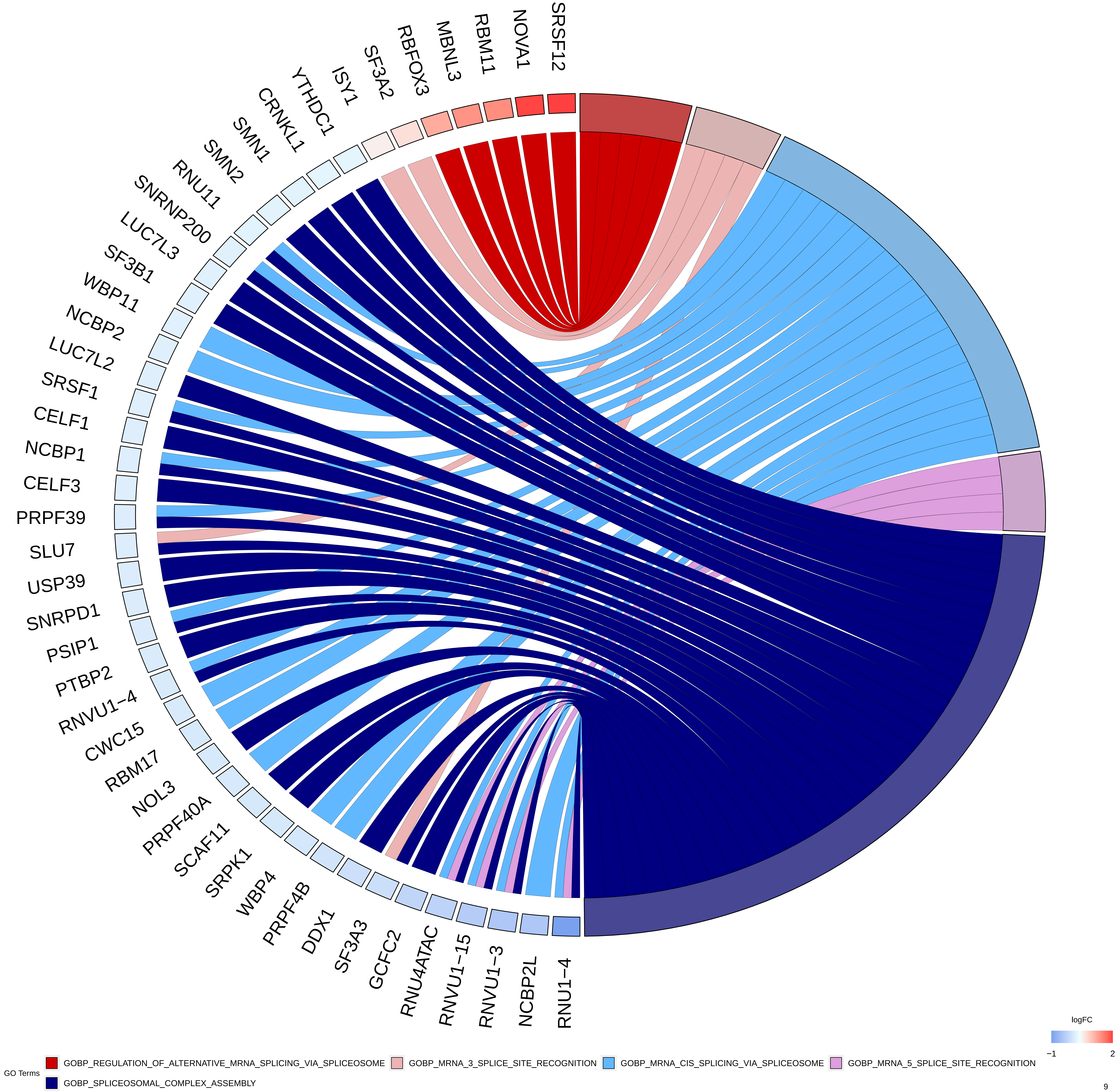

Supplementary Figure 6: GOChord Splicing FSHD_group2

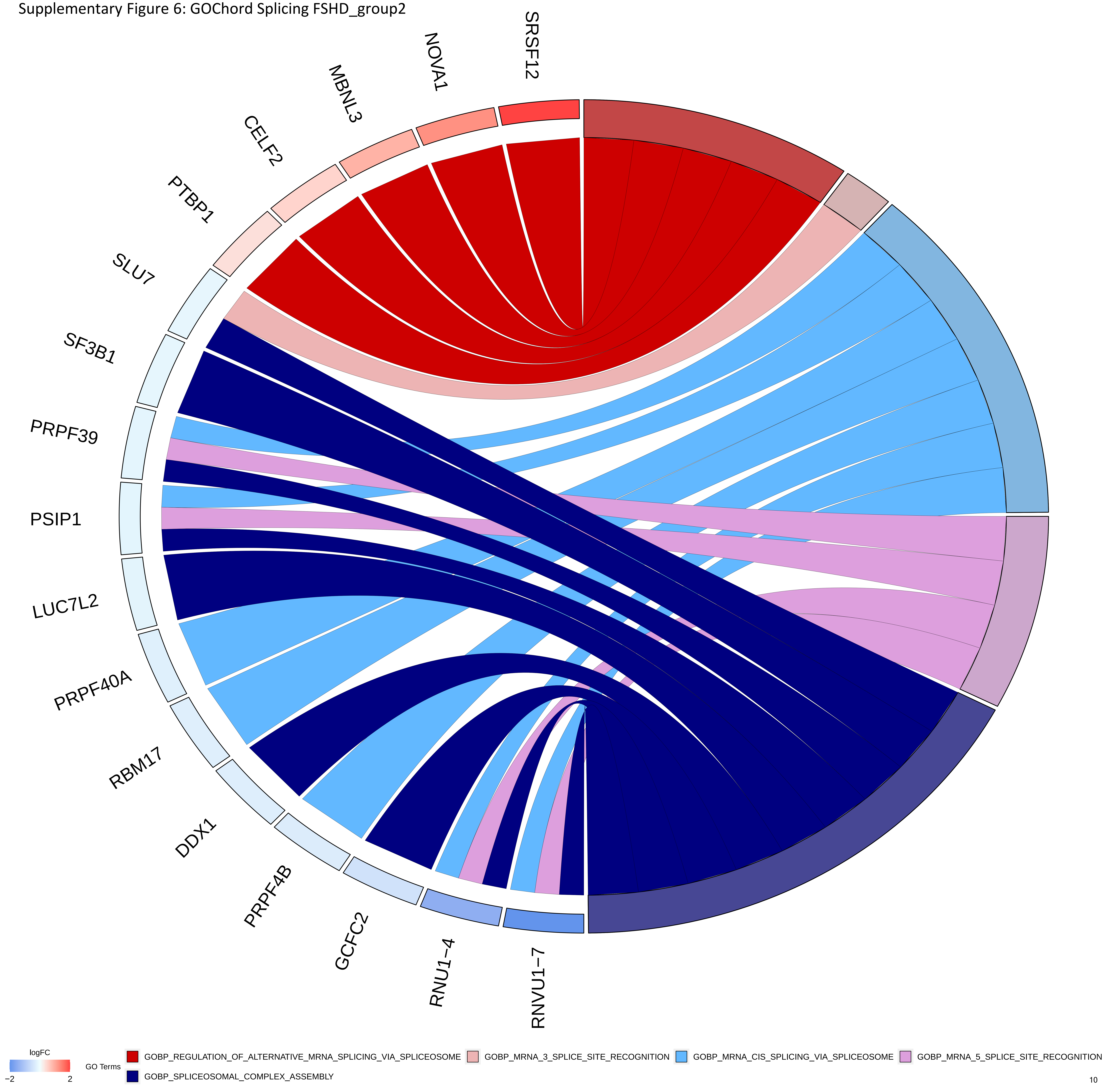

Supplementary Tables

Meta-analysis towards FSHD reveals misregulation of neuromuscular junction, nuclear envelope, and spliceosome

Teresa Schätzl[1], Vanessa Todorow[2], Lars Kaiser[1], Helga Weinschrott[1], Benedikt Schoser[2], Hans-Peter Deigner[1,3,4], Peter Meinke[2], Matthias Kohl[1]

[1] Institute of Precision Medicine, Furtwangen University, Germany

[2] Friedrich-Baur-Institute at the Department of Neurology, LMU University Hospital, Ludwig Maximilian University, Munich, Germany

[3] Faculty of Science, Eberhard-Karls-University Tuebingen, Auf Der Morgenstelle 8, 72076, Tübingen, Germany

[4] EXIM Department, Fraunhofer Institute IZI (Leipzig), Schillingallee 68, 18057, Rostock, Germany

Table of contents

1. Overview of Datasets 2

2. Checklists 11

3. NMJ Associated Genes 15

Supplementary Table 1: Overview of Datasets

	Dataset	year	technology	patients/controls	GEO ID: FSHD	sex	age	D4Z4 *1	tissue *2	path. features *3	ad.p<0.05 *4
CELL LINES Microarrays	GSE26061	2011	Affymetrix Human Exon 1.0 ST Array	3 x FSHD1 MB + corr.* MT<br>2 x FSHD2 MB + corr. MT<br>3 x CTRL MB + corr. MT	MB: GSM639904<br>MT: GSM639907	m	5	1	Q	N/A	→ FSHD *vs* CTRL<br>/
					MB: GSM639905<br>MT: GSM639908	f	71	7	Q	N/A	
					MB: GSM639906<br>MT: GSM639909	f	55	7	Q	N/A	
					FSHD2 MB: GSM639910<br>MT: GSM639912	m	12	>38	Q	N/A	
					FSHD2 MB: GSM639911<br>MT: GSM639913	m	17	>38	Q	N/A	
	GSE26145	2011	Affymetrix Human Exon 1.0 ST Array	3 x FSHD1 MB<br>2 x corr. MT<br>1 x new MT<br>3 x CTRL MB<br>+ corr. MT	MB: GSM641863<br>MT: GSM641864	f	45	7	Q	N/A	→ FSHD *vs* CTRL<br>/
					MB: GSM641865<br>MT: GSM641866	f	22	5	R	N/A	
					MB: GSM641867	m	13	4	Q	N/A	
					MT: GSM641868	f	14	2	D	N/A	
BIOPSIES Microarrays	GSE10760 *5	2008	Affymetrix Human Genome U133A Array<br>+<br>Affymetrix Human Genome U133B Array	19 x FSHD1<br>30 x CTRL	U133A: GSM271541<br>U133B: GSM271688	m	38	7	VL	N/A	FSHD vs CTRL<br>4459
					U133A: GSM271542<br>U133B: GSM271689	m	39	8	VL	N/A	
					U133A: GSM271543<br>U133B: GSM271690	m	56	9	VL	Path.sc.: 5	
					U133A: GSM271544<br>U133B: GSM271691	m	44	9	VL	N/A	
					U133A: GSM271545<br>U133B: GSM271692	m	25	6	VL	N/A	
					U133A: GSM271546<br>U133B: GSM271693	m	29	8	VL	Path.sc.: 4	
					U133A: GSM271547<br>U133B: GSM271694	m	73	5	VL	N/A	
					U133A: GSM271548<br>U133B: GSM271695	m	57	4	VL	Path.sc.: 4	

*1 When EcoRI/BlnI fragments were given instead of D4Z4 repeat number, D4Z4 repeats were calculated using the following formula [12]: $Repeats = \frac{EcoRi\ fragment\ size\ in\ kb - 5kb\ flanking\ sequence}{3.3kb}$

*2 Tissue: Q = Quadriceps femoris; R = Rhomboideus major; D = Deltoideus; VL = Vastus lateralis; B = Biceps brachii; P = Paravertebral; TA =Tibialis anterior; G = Gatrocnemius; H = Hamstring

*3 The report of pathologic features varies depending on the dataset; In all datasets CSS is based on the 10-grade Clinical Severity Score developed by Ricci et al. (1999) [3]

*4 The calculation of the adj. p-value of the individual datasets refers to the *vote-counting* approach of the meta-analysis [4]

*5 *GSE10760.* Data on age (yr), gender, EcoRI/BlnI fragments, path. score and disease severity were kindly provided after email request

BIOPSIES Microarrays	GSE10760	2008	Affymetrix Human Genome U133A Array + Affymetrix Human Genome U133B Array	19 x FSHD1<br>30 x CTRL	U133A: GSM271549<br>U133B: GSM271696	m	48	8	VL	N/A	FSHD vs CTRL<br>4459
					U133A: GSM271550<br>U133B: GSM271697	m	41	4	VL	Path.sc.: 9	
					U133A: GSM271551<br>U133B: GSM271698	f	63	N/A	VL	N/A	
					U133A: GSM271552<br>U133B: GSM271699	f	40	3	VL	N/A	
					U133A: GSM271553<br>U133B: GSM271700	f	60	9	VL	N/A	
					U133A: GSM271554<br>U133B: GSM271701	f	36	6	VL	Path.sc.: 2; Disease Severity: 5	
					U133A: GSM271555<br>U133B: GSM271702	f	37	7	VL	N/A	
					U133A: GSM271556<br>U133B: GSM271703	f	55	7	VL	Disease Severity: 7	
					U133A: GSM271557<br>U133B: GSM271704	f	36	6	VL	Path.sc.: 3; Disease Severity: 7	
					U133A: GSM271558<br>U133B: GSM271705	f	30	N/A	VL	N/A	
					U133A: GSM271559<br>U133B: GSM271706	f	17	N/A	VL	N/A	
	GSE15090	2009	Affymetrix Human Genome U133 Plus 2.0 Array	5 x FSHD1 *6<br>5 x CTRL	GSM377456	m	49	5	B	F,S,P,A *7	FSHD vs CTRL<br>/
					GSM377459	m	30	2	D	F,S,P,A,H	
					GSM377462	f	14	3	D	F,S,D	
					GSM377465	m	11	5	B	F,S,P,L	
					GSM377468	m	38	8	B	S,P	
	GSE36398	2012	Affymetrix Human Gene 1.0 ST Array	13 x FSHD1 *8<br>12 x CTRL (unaffected first-degree relatives)	GSM892342	f	40	15	B	Biceps strengths: 4+/5 *9	FSHD vs CTRL<br>/
					GSM892345	f	18	7	B	Biceps strengths: 5/5	
					GSM892349	f	31	6	B	Biceps strengths: 4+/5	
					GSM892353	f	22	4	B	Biceps strengths: 4+/5	
					GSM892355	m	49	4	B	Biceps strengths: 4+/5	
					GSM892361	f	42	3	B	Biceps strengths: 4/5	

*5 *GSE10760.* Data on age (yr), gender, EcoRI/BlnI fragments, path. score and disease severity were kindly provided after email request.

*6 *GSE15090.* Asymptomatic samples were excluded.

*7 *GSE15090.* Muscle weakness (+ other features): A = ankle; D = depression; F = face; H = hearing loss; L = swallowing problem; P = pelvic girdle; S = shoulder girdle [5]

*8 *GSE36398.* Biopsies were taken from both the biceps and deltoid muscle of the same individual for further analysis of more affected versus less affected muscle tissue [6]; In the meta-analysis, only the biceps samples were included

*9 *GSE36398.* Muscle strength of biopsied muscles is presented using a modified MRC scale, where 5/5 is full strength [6]

BIOPSIES Microarrays	GSE36398	2012	Affymetrix Human Gene 1.0 ST Array	13 x FSHD1<br>12 x CTRL (unaffected first-degree relatives)	GSM892365	f	36	5	B	Biceps strengths: 4+/5	FSHD vs CTRL<br>/
					GSM892369	m	65	5	B	Biceps strengths: 4+/5	
					GSM892373	m	66	7	B	Biceps strengths: 4+/5	
					GSM892377	m	28	5	B	Biceps strengths: 5/5	
					GSM892381	f	82	6	B	Biceps strengths: 4+/5	
					GSM892383	f	59	6	B	Biceps strengths: 4+/5	
					GSM892387	f	71	7	B	Biceps strengths: 4+/5	
	GSE26852	2012	Illumina HumanHT-12 V3.0 expression beadchip	8 x FSHD1 *10<br>7 x CTRL	GSM661131	m	31	N/A	Q	T2-STIR – *11	→ FSHD vs CTRL<br>↘ Total:<br>/ *12<br>↘ T2-STIR–:<br>/<br>↘ T2-STIR+:<br>3489
					GSM661132	m	N/A	N/A	Q	T2-STIR –	
					GSM661134	f	N/A	N/A	Q	T2-STIR –	
					GSM661135	m	N/A	N/A	Q	T2-STIR –	
					GSM661137	m	31	N/A	P	T2-STIR +	
					GSM661138	m	N/A	N/A	B	T2-STIR +	
					GSM661139	f	N/A	N/A	Q	T2-STIR +	
					GSM661141	f	N/A	N/A	B	T2-STIR +	
CELL LINES RNA-Seq	GSE56787 *13	2014	Illumina HiSeq 2500	2 x FSHD1 MB<br>+ corr. MT<br><br>2 x FSHD2 MB<br>+ corr. MT<br>1 x FSHD2 MT (separate)<br><br>2 x CTRL MB<br>+ corr. MT<br>1 x CTRL MT (separate)	MB: GSM1412732<br>MT: GSM1412733	m	33	6	Q	CSS: 6; Path.sc.: 5 *14	→ FSHD *vs* CTRL<br>/
					MB: GSM1412734<br>MT: GSM1412735	f	30	5	Q	CSS: 3; Path.sc.: 3	
					FSHD2 – MB: GSM1412728<br>MT: GSM1412729	f	56	N/A	Q	N/A	
					FSHD2 – MB: GSM1412730<br>MT: GSM1412731	f	N/A	N/A	N/A	CSS: 3; Path.sc.: 3	
					FSHD2 – MT: GSM1412727	m	26	N/A	Q	CSS: 6; Path.sc.: 4	

*10 *GSE26852.* Assignment of the data considering the associated publication only allows the following conclusion: Because 2 biopsies (GSM661131, quadriceps *and* GSM661137, paravertebral) were taken from the same individual and this is noted in the publication, age (31) and sex (m) can be related to this individual; all other data cannot be clearly allocated, but detailed descriptions about the STIR+ samples (marked/moderate myopathic changes) and STIR- samples (mild/minimal myopathic changes) can be found in Tasca et al. (2012) [7]

*11 *GSE26852.* By muscle Magnetic Resonance Imaging (MRI) it was observed that T2-short tau inversion recovery (T2-STIR) sequences identify two different conditions in which each muscle can be found before the irreversible dystrophic alteration, marked as T1-weighted sequence hyperintensity, takes place: T1-weighted normal/T2-STIR normal *and* T1-weighted normal/T2-STIR hyperintense [7]

*12 *GSE26852.* If all data are included in the analysis, no significant results are obtained; the same applies if only the T2-STIR*minus* samples are considered; if the T2-STIR*minus* samples are excluded from the analysis and only the T2-STIR*plus* samples are used, 3489 significant results are obtained. The latter were included in the meta-analysis; for all other microarrays without significant results, no such additional information is available

*13 *GSE56787.* The dataset contains data from both biopsies and cell lines and is therefore split in the meta-analysis (see BIOPSIES RNA-Seq for the other part of the data)

*14 *GSE56787.* Pathology score: A twelve point scale (0 = normal muscle and 12 = end-stage muscle) was used for histopathological grading of the sample based on: degree of muscle fiber variability, extent of central nucleation, presence of muscle fiber necrosis/regeneration and extent of interstitial fibrosis [8]

CELL LINES RNA-Seq	GSE123468	2018	Illumina HiSeq 2500	2 x FSHD1 [*15]<br>x 2 runs (MB → MT)<br>in triplicates<br><br>2 x CTRL (sister each)<br>x 2 runs<br>in triplicates	GSM3504589 (MB,MT)	f	22	4	B	Biceps strengths: 4+/5 [*16]	Family 12<br>→ FSHD *vs* CTRL<br>347
					GSM3504590 (MB,MT)	f	22	4	B	Biceps strengths: 4+/5	
					GSM3504591 (MB,MT)	f	22	4	B	Biceps strengths: 4+/5	
					GSM3504595 (MB,MT)	f	56	5	B	Biceps strengths: 4+/5	Family 16<br>→ FSHD *vs* CTRL<br>1579
					GSM3504596 (MB,MT)	f	56	5	B	Biceps strengths: 4+/5	
					GSM3504597 (MB,MT)	f	56	5	B	Biceps strengths: 4+/5	
BIOPSIES RNAseq	GSE56787 [*17]	2014	Illumina HiSeq 2500	9 x FSHD1<br>6 x FSHD2<br>9 x CTRL	GSM1412707	m	47	6	Q	CSS: 0; Path.sc.: 2 [*14]	FSHD vs CTRL<br>/
					GSM1412714	f	45	4	Q	CSS: 5; Path.sc.: N/A	
					GSM1412715	f	55	3	Q	CSS: 6; Path.sc.: 4	
					GSM1412716	m	33	6	Q	CSS: 6; Path.sc.: 5	
					GSM1412717	m	52	5	Q	CSS: 6; Path.sc.: N/A	
					GSM1412718	f	30	5	Q	CSS: 3; Path.sc.: 3	
					GSM1412719	f	61	3	Q	CSS: 6; Path.sc.: 6	
					GSM1412720	f	26	3	Q	CSS: 7; Path.sc.: 5	
					GSM1412721	m	48	4	Q	CSS: 3; Path.sc.: 4	
					FSHD2: GSM1412708	m	42	N/A	Q	CSS: 5; Path.sc.: 2	
					FSHD2: GSM1412709	m	34	N/A	Q	CSS: 6; Path.sc.: 3	
					FSHD2: GSM1412710	m	26	N/A	Q	CSS: 6; Path.sc.: 4	
					FSHD2: GSM1412711	m	50	N/A	Q	CSS: 5; Path.sc.: 5	
					FSHD2: GSM1412712	f	56	N/A	Q	CSS: 7; Path.sc.: 3	
					FSHD2: GSM1412713	m	59	N/A	Q	CSS: 2; Path.sc.: 2	

*14 *GSE56787*. Pathology score: A twelve point scale (0 = normal muscle and 12 = end-stage muscle) was used for histopathological grading of the sample based on: degree of muscle fiber variability, extent of central nucleation, presence of muscle fiber necrosis/regeneration and extent of interstitial fibrosis [8]

*15 *GSE123468*. Cell lines are immortalized [9]

*16 *GSE123468*. Muscle strength is presented using a modified Medical Research Council (MRC) scale where 5/5 is full strength [9,10]

*17 *GSE56787*. The dataset contains data from both biopsies and cell lines and is therefore split in the meta-analysis (see CELL LINES RNA-Seq for the other part of the data)

BIOPSIES RNA-Seq	GSE115650	2018	Illumina HiSeq 2500	30 x FSHD1 *18<br>3 x FSHD2<br>9 x CTRL	GSM3186670	m	59	5	TA	CSS: 5; Path.sc.: 7; Inflam.: 0 *19<br>STIR: 4; T1: 1; Fat: 6	FSHD vs CTRL<br>1175
					GSM3186671	m	58	4	G	CSS: 6; Path.sc.: 4; Inflam.: 2<br>STIR: 3; T1: 0; Fat: 21	
					GSM3186672	f	51	6	Q	CSS: 6; Path.sc.: 6; Inflam.: 1<br>STIR: 2; T1: 1; Fat: 21	
					GSM3186673	m	42	9	G	CSS: 2; Path.sc.: 10; Inflam.: 3<br>STIR: 2; T1: 4; Fat: 32	
					GSM3186675	m	25	6	G	CSS: 0; Path.sc.: 3; Inflam.: 0<br>STIR: 0; T1: 0; Fat: 5	
					GSM3186676	m	61	6	G	CSS: 6; Path.sc.: 0; Inflam.: 0<br>STIR: 0; T1: 0; Fat: 6	
					GSM3186677	f	67	7	TA	CSS: 3; Path.sc.: 5; Inflam.: 1<br>STIR: 0; T1: 0; Fat: 13	
					GSM3186678	f	57	5	TA	CSS: 6; Path.sc.: 2; Inflam.: 0<br>STIR: 2; T1: 0; Fat: 6	
					GSM3186679	f	64	7	H	CSS: 5; Path.sc.: 10; Inflam.: 3<br>STIR: 2; T1: 0; Fat: 11	
					GSM3186680	m	56	4	Q	CSS: 5; Path.sc.: 6; Inflam.: 0<br>STIR: 0; T1: 3; Fat: 45	
					GSM3186681	f	54	6	TA	CSS: 6; Path.sc.: 5; Inflam.: 0<br>STIR: 0; T1: 0; Fat: 11	
					GSM3186682	m	49	6	TA	CSS: 4; Path.sc.: 5; Inflam.: 0<br>STIR: 0; T1: 0; Fat: 6	
					GSM3186684	m	60	8	G	CSS: 7; Path.sc.: 5; Inflam.: 0<br>STIR: 2; T1: 0; Fat: 11	
					GSM3186685	m	60	5	Q	CSS: 8; Path.sc.: 8; Inflam.: 0<br>STIR: 1; T1: 2; Fat: 15	
					GSM3186687	f	53	4	G	CSS: 2; Path.sc.: 7; Inflam.: 0<br>STIR: 2; T1: 1; Fat: 16	
					GSM3186688	m	32	5	G	CSS: 6; Path.sc.: 4; Inflam.: 0<br>STIR: 2; T1: 0; Fat: 8	
					GSM3186689	m	48	9	G	CSS: 6; Path.sc.: 4; Inflam.: 0<br>STIR: 1; T1: 0; Fat: 4	
					GSM3186690	m	65	8	G	CSS: 7; Path.sc.: 7; Inflam.: 1<br>STIR: 2; T1: 1; Fat: 17	

*18 *GSE115650.* 1 Mosaic sample was excluded

*19 *GSE115650.* Pathologic severity score based on a 12-point scale (0–3 score to each of the four major histologic features: 1 = variability in fiber size, 2 = percent of centrally located nuclei, 3 = interstitial fibrosis, 4 = muscle fiber necrosis/regeneration/inflammation); Pathologic scores of 1–4 = mild, 5–8 = moderate and 9–12 = severe; STIR intensity was rated on a four-point scale as stage 0 = normal appearance; stage 1 = very mild diffuse elevation; stage 2 = mild diffuse elevation with areas of moderate signal elevation, less than 30% of the muscle volume; stage 3 = moderate areas of increased signal intensity, 30–60% volume; stage 4 = moderate/severe involvement of entire muscle [11]

BIOPSIES RNA-Seq	GSE115650	2018	Illumina HiSeq 2500	30 x FSHD1<br>3 x FSHD2<br>9 x CTRL		GSM3186697	m	20	5	Q	CSS: 3; Path.sc.: 2; Inflam.: 0<br>STIR: 0; T1: 0; Fat: 4	FSHD vs CTRL<br>1175
						GSM3186698	f	52	5	TA	CSS: 6; Path.sc.: N/A; Inflam.: N/A<br>STIR: 2; T1: 1; Fat: 23	
						GSM3186699	f	67	9	Q	CSS: 3; Path.sc.: 2; Inflam.: 0<br>STIR: 0; T1: 0; Fat: 6	
						GSM3186700	f	42	5	G	CSS: 2; Path.sc.: 5; Inflam.: 1<br>STIR: 1; T1: 1; Fat: 7	
						GSM3186701	f	62	5	TA	CSS: 2; Path.sc.: 4; Inflam.: 0<br>STIR: 2; T1: 0; Fat: 16	
						GSM3186702	f	65	7	TA	CSS: 4; Path.sc.: 4; Inflam.: 0<br>STIR: 1; T1: 0; Fat: 6	
						GSM3186707	m	55	8	TA	CSS: 5; Path.sc.: 7; Inflam.: 0<br>STIR: 0; T1: 0; Fat: 5	
						GSM3186708	m	51	5	G	CSS: 6; Path.sc.: 4; Inflam.: 0<br>STIR: 0; T1: 0; Fat: 11	
						GSM3186709	m	59	6	G	CSS: 5; Path.sc.: N/A; Inflam.: N/A<br>STIR: 2; T1: 3; Fat: 43	
						GSM3186710	m	42	4	TA	CSS: 5; Path.sc.: 7; Inflam.: 1<br>STIR: 2; T1: 1; Fat: 9	
						GSM3186711	f	62	4	TA	CSS: 6; Path.sc.: 1; Inflam.: 0<br>STIR: 1; T1: 0; Fat: 1	
						GSM3186712	m	75	6	Q	CSS: 7; Path.sc.: 2; Inflam.: 0<br>STIR: 2; T1: 1; Fat: 7	
					FSHD2	GSM3186674	m	62	9	TA	CSS: 6; Path.sc.: 8; Inflam.: 1<br>STIR: 2; T1: 4; Fat: 54	
						GSM3186683	m	56	26	G	CSS: 3; Path.sc.: 8; Inflam.: 1<br>STIR: 2; T1: 0; Fat: 5	
						GSM3186686	m	31	12	G	CSS: 6; Path.sc.: 8; Inflam.: 2<br>STIR: 2; T1: 1; Fat: 22	
	EGAD00001008337	2022	Illumina HiSeq 4000<br>Illumina NovaSeq 6000<br>NextSeq 500	30 x FSHD1 *20<br>7 x FSHD2<br>24 x CTRL		FSHD_02_VL_re	f	52	8	VL	*21<br>CSS: 7; Path.sc.: 6; Inflam.: 2<br>TIRM: Neg; Fat: 58	FSHD vs CTRL<br>7326
						FSHD_13_VL_re	m	55	6	VL	CSS: 5; Path.sc.: 5; Inflam.: 1<br>TIRM: Pos; Fat: 0	
						FSHD_01_VL	m	56	8	VL	CSS: 6; Path.sc.: 3; Inflam.: 0<br>TIRM: Neg; Fat: 3	

*20 *EGAD00001008337*. Exclusion of 2 mosaic samples and 2 asymptomatic samples

*21 *EGAD00001008337*. Turbo Inversion Recovery sequences (TIRM) positivity was a binary determination based on the T2 signal. Muscle biopsies acquired from TIRM hyperintense muscles or areas are denoted as TIRM^{POS}, whereas muscle biopsies acquired from TIRM negative muscles or areas are TIRM^{NEG} [12]

**BIOSPIES RNA-Seq**	**EGAD00001008337**	2022	Illumina HiSeq 4000<br>Illumina NovaSeq 6000<br>NextSeq 500	30 x FSHD1<br>7 x FSHD2<br>24 x CTRL	FSHD_02_VL	f	52	8	VL	CSS: 7; Path.sc.: 6; Inflam.: 2<br>TIRM: Neg; Fat: 58	FSHD vs CTRL<br>7326
					FSHD_02_TA	f	52	8	TA	CSS: 7; Path.sc.: 7; Inflam.: 0<br>TIRM: Neg; Fat: 20	
					FSHD_04_TA	f	44	5	TA	CSS: 3; Path.sc.: 4; Inflam.: 0<br>TIRM: Neg; Fat: 10	
					FSHD_07_VL	m	52	7	VL	CSS: 2; Path.sc.: 4; Inflam.: 1<br>TIRM: Neg; Fat: 0	
					FSHD_07_TA	m	52	7	TA	CSS: 2; Path.sc.: 6; Inflam.: 0<br>TIRM: Neg; Fat: 10	
					FSHD_08_VL	m	49	8	VL	CSS: 4; Path.sc.: 1; Inflam.: 0<br>TIRM: Neg; Fat: 0	
					FSHD_08_TA	m	49	8	TA	CSS: 4; Path.sc.: 2; Inflam.: 0<br>TIRM: Neg; Fat: 1	
					FSHD_11_VL	m	59	10	VL	CSS: 2; Path.sc.: 1; Inflam.: 0<br>TIRM: Neg; Fat: 5	
					FSHD_11_TA	m	59	10	TA	CSS: 2; Path.sc.: 2; Inflam.: 1<br>TIRM: Neg; Fat: 6	
					FSHD_12_VL	f	59	9	VL	CSS: 3; Path.sc.: 3; Inflam.: 0<br>TIRM: Neg; Fat: 1	
					FSHD_12_TA	f	59	9	TA	CSS: 3; Path.sc.: 4; Inflam.: 0<br>TIRM: Neg; Fat: 5	
					FSHD_13_VL	m	55	6	VL	CSS: 5; Path.sc.: 5; Inflam.: 1<br>TIRM: Pos; Fat: 0	
					FSHD_13_VL2	m	55	6	VL	CSS: 5; Path.sc.: 4; Inflam.: 0<br>TIRM: Neg; Fat: 2	
					FSHD_14_VL	f	56	7	VL	CSS: 8; Path.sc.: 4; Inflam.: 0<br>TIRM: Neg; Fat: 5	
					FSHD_14_TA	f	56	7	TA	CSS: 8; Path.sc.: 3; Inflam.: 1<br>TIRM: Pos; Fat: 69	
					FSHD_16_VL	m	28	3	VL	CSS: 6; Path.sc.: N/A; Inflam.: N/A<br>TIRM: Neg; Fat: 5	
					FSHD_17_VL	m	29	3	VL	CSS: 7; Path.sc.: N/A; Inflam.: N/A<br>TIRM: Neg; Fat: 7	
					FSHD_18_VL	m	42	8	VL	CSS: 1; Path.sc.: N/A; Inflam.: N/A<br>TIRM: Neg; Fat: 6	
					FSHD_19_VL	m	63	5	VL	CSS: 7; Path.sc.: N/A; Inflam.: N/A<br>TIRM: Pos; Fat: 25	
					FSHD_20_VL	m	40	8	VL	CSS: 3; Path.sc.: N/A; Inflam.: N/A<br>TIRM: Neg; Fat: 5	

BIOPSIES RNA-Seq	EGAD00001008337	2022	Illumina HiSeq 4000<br>Illumina NovaSeq 6000<br>NextSeq 500	30 x FSHD1<br>7 x FSHD2<br>24 x CTRL		FSHD_21_VL	f	74	8	VL	CSS: 2; Path.sc.: N/A; Inflam.: N/A<br>TIRM: Neg; Fat: 7	FSHD vs CTRL<br>7326
						FSHD_22_VL	f	55	5	VL	CSS: 6; Path.sc.: N/A; Inflam.: N/A<br>TIRM: Neg; Fat: 6	
						FSHD_24_VL	f	45	5	VL	CSS: 6; Path.sc.: N/A; Inflam.: N/A<br>TIRM: Neg; Fat: 12	
						FSHD_25_VL	m	20	5	VL	CSS: 4; Path.sc.: N/A; Inflam.: N/A<br>TIRM: Neg; Fat: 8	
						FSHD_26_VL	f	56	5	VL	CSS: 8; Path.sc.: N/A; Inflam.: N/A<br>TIRM: Pos; Fat: 12	
						FSHD_27_VL	m	49	7	VL	CSS: 0; Path.sc.: N/A; Inflam.: N/A<br>TIRM: Neg; Fat: 5	
						FSHD_28_VL	m	67	9	VL	CSS: 7; Path.sc.: N/A; Inflam.: N/A<br>TIRM: Neg; Fat: 13	
					FSHD2	FSHD_03_VL	f	48	N/A	VL	CSS: 6; Path.sc.: 4; Inflam.: 1<br>TIRM: Neg; Fat: 5	
						FSHD_03_TA	f	48	N/A	TA	CSS: 6; Path.sc.: 1; Inflam.: 0<br>TIRM: Neg; Fat: 6	
						FSHD_06_VL	f	57	N/A	VL	CSS: 6; Path.sc.: 2; Inflam.: 0<br>TIRM: Neg; Fat: 28	
						FSHD_06_TA	f	57	N/A	TA	CSS: 6; Path.sc.: 4; Inflam.: 0<br>TIRM: Neg; Fat: 6	
						FSHD_09_VL1	m	50	N/A	VL	CSS: 8; Path.sc.: 10; Inflam.: 1<br>TIRM: Pos; Fat: 53	
						FSHD_09_VL2	m	50	N/A	VL	CSS: 8; Path.sc.: 8; Inflam.: 0<br>TIRM: Neg; Fat: 57	
						FSHD_10_TA	m	61	N/A	TA	CSS: 6; Path.sc.: 7; Inflam.: 0<br>TIRM: Neg; Fat: 64	
DUX4 model RNA-Seq	GSE138768	2021	Illumina HiSeq 2500	4x DUX4 over expression<br>4x Empty vector		GSM4119029	DUX4 cell model:<br><br>Generation of human skeletal micro-muscles expressing inducible DUX4				DUX4 over-expression	FSHD vs CTRL<br>2843
						GSM4119030					DUX4 over-expression	
						GSM4119031					DUX4 over-expression	
						GSM4119032					DUX4 over-expression	
						GSM4119033					Empty vector	
						GSM4119034					Empty vector	
						GSM4119035					Empty vector	
						GSM4119036					Empty vector	

References

1. Butz, M. *et al.* Facioscapulohumeral muscular dystrophy: Phenotype-genotype correlation in patients with borderline D4Z4 repeat numbers. *J Neurol* **250**, 932–937 (2003).
2. van der Maarel, S. M. *et al.* De novo facioscapulohumeral muscular dystrophy: Frequent somatic mosaicism, sex-dependent phenotype, and the role of mitotic transchromosomal repeat interaction between chromosomes 4 and 10. *Am J Hum Genet* **66**, 26–35 (2000).
3. Ricci, E. *et al.* Progress in the molecular diagnosis of facioscapulohumeral muscular dystrophy and correlation between the number of KpnI repeats at the 4q35 locus and clinical phenotype. *Ann Neurol* **45**, 751–757 (1999).
4. Ramasamy, A., Mondry, A., Holmes, C. C. & Altman, D. G. Key issues in conducting a meta-analysis of gene expression microarray datasets. *PLoS Med* **5**, 1320–1332 (2008).
5. Arashiro, P. *et al.* Transcriptional regulation differs in affected facioscapulohumeral muscular dystrophy patients compared to asymptomatic related carriers. *Proceedings of the National Academy of Sciences* **106**, 6220–6225 (2009).
6. Rahimov, F. *et al.* Transcriptional profiling in facioscapulohumeral muscular dystrophy to identify candidate biomarkers. *Proc Natl Acad Sci U S A* **109**, 16234–16239 (2012).
7. Tasca, G. *et al.* Different molecular signatures in magnetic resonance imaging-staged facioscapulohumeral muscular dystrophy muscles. *PLoS One* **7**, (2012).
8. Yao, Z. *et al.* DUX4-induced gene expression is the major molecular signature in FSHD skeletal muscle. *Hum Mol Genet* **23**, 5342–5352 (2014).
9. Banerji, C. R. S. *et al.* Dynamic transcriptomic analysis reveals suppression of PGC1α/ERRα drives perturbed myogenesis in facioscapulohumeral muscular dystrophy. *Hum Mol Genet* **28**, 1244–1259 (2019).
10. Homma, S. *et al.* A unique library of myogenic cells from facioscapulohumeral muscular dystrophy subjects and unaffected relatives: Family, disease and cell function. *European Journal of Human Genetics* **20**, 404–410 (2012).
11. Wang, L. H. *et al.* MRI-informed muscle biopsies correlate MRI with pathology and DUX4 target gene expression in FSHD. *Hum Mol Genet* **28**, 476–486 (2019).
12. van den Heuvel, A. *et al.* Facioscapulohumeral dystrophy transcriptome signatures correlate with different stages of disease and are marked by different MRI biomarkers. *Sci Rep* **12**, (2022).

Supplementary Table 2: Checklists

2a) Conducting Meta-Analysis of Microarray Datasets

Step	Action
**Identify suitable microarray studies (Issue 1)**	
✔ **Supplementary Notes 1-2, Supplementary Table 1**	
1	Formulate objectives and a review protocol.
2	Define inclusion-exclusion criteria and suitable keywords.
3	Perform literature search using the keywords on the Web sites listed in Table 2.
4	Search public microarray repositories listed in Table 2.
5	Contact collaborators and experts in the field to help find published and unpublished data.
6	Search the reference section of retrieved studies for other relevant studies.
7	Check the selected study against inclusion-exclusion criteria.
**Extract the data from studies (Issue 2)**	
✔ **Supplementary Notes 1**	
8	Scan the literature to identify FLEO data (e.g., CEL, GPR files).
9	If the main text does not contain a link to FLEO data, search the repositories and group/lab's Web pages. If unsuccessful, write to the authors.
10	If multiple publications use overlapping data, identify the most comprehensive one. Combine any training and validation dataset together
**Prepare the individual datasets (Issue 3)**	
✔ **Supplementary Notes 1-2, Supplementary Table 1**	
11	Identify and remove any arrays with poor quality.
12	Preprocess the FLEO data into a GEDM.
13	Check for batch effects among arrays, especially in large studies.
14	Filter out any probes with poor spot quality in the arrays (optional).
15	Aggregate any technical replicates.
16	Check that the processed expression values from multiple platforms are compatible.
**Annotate the individual datasets (Issue 4)**	
✔ **Supplementary Notes 1**	
17	Identify either (a) the probe sequence or (b) the most sequence-specific probe annotation information.
18	Either (a) cluster the probe sequences or (b) map the most sequence-specific probe annotation to a gene-level identifier. Use the same mapping build for all datasets.
**Resolve the many-to-many relationship between probes and genes (Issue 5)**	
✔ **Supplementary Notes 1**	
19	Discard any probe that does not map to any GeneID.
20	For every GeneID within a study, calculate the study-specific estimate(s).
21	If a probe maps to multiple GeneIDs within a study, "expand" it by replacing it with a new record for each GeneID with the same study-specific estimate(s) or expression profile.
22	For GeneIDs with multiple records within a study, "summarize" them by either selecting one of the records or by aggregating them
**Combine the study-specific estimates (Issue 6)**	
✔ **Supplementary Notes 1**	
23	For every GeneID, identify the studies that provide usable information. Optionally, discard any GeneID that is not found in at least a prespecified number of studies.
24	For every GeneID, combine the study-specific estimates across the studies using a meta-analytic technique. Record the resulting summary statistic(s).
25	Calculate the nominal p-value of the summary statistic(s) for every GeneID & adjust for multiple testing
**Analyze, present, and interpret results (Issue 7)**	
✔ **Supplementary Notes 3-4, Supplementary Figures 1-3, Figures 1-5**	
26	Examine the sensitivity of results to individual studies with a leave-one-out analysis and by varying the selections made (e.g., type of data available).
27	Present the summary statistics graphically (e.g., forest plot) for genes of interest.
28	Analyze findings using computational tools (e.g., gene set enrichment analysis).
29	If possible, validate using an alternative technology and/or different samples.
30	Consider strength of evidence, limitations, and generalizability of current findings.

Reference:

Ramasamy, A., Mondry, A., Holmes, C. C. & Altman, D. G. Key issues in conducting a meta-analysis of gene expression microarray datasets. *PLoS Med* 5, 1320-1332 (2008)

2b) PRISMA 2020 Checklist

Section and Topic	Item #	Checklist item	Location where item is reported
**TITLE**			
Title	1	Identify the report as a systematic review.	p.1
**ABSTRACT**			
Abstract	2	See the PRISMA 2020 for Abstracts checklist.	p.1
**INTRODUCTION**			
Rationale	3	Describe the rationale for the review in the context of existing knowledge.	Sec Intro
Objectives	4	Provide an explicit statement of the objective(s) or question(s) the review addresses.	Notes 1
**METHODS**			
Eligibility criteria	5	Specify the inclusion and exclusion criteria for the review and how studies were grouped for the syntheses.	Notes 1
Information sources	6	Specify all databases, registers, websites, organisations, reference lists and other sources searched or consulted to identify studies. Specify the date when each source was last searched or consulted.	Notes 1
Search strategy	7	Present the full search strategies for all databases, registers and websites, including any filters and limits used.	Notes 1
Selection process	8	Specify the methods used to decide whether a study met the inclusion criteria of the review, including how many reviewers screened each record and each report retrieved, whether they worked independently, and if applicable, details of automation tools used in the process.	Notes 1
Data collection process	9	Specify the methods used to collect data from reports, including how many reviewers collected data from each report, whether they worked independently, any processes for obtaining or confirming data from study investigators, and if applicable, details of automation tools used in the process.	Notes 1
Data items	10a	List and define all outcomes for which data were sought. Specify whether all results that were compatible with each outcome domain in each study were sought (e.g. for all measures, time points, analyses), and if not, the methods used to decide which results to collect.	Notes 1-2 Table 1
	10b	List and define all other variables for which data were sought (e.g. participant and intervention characteristics, funding sources). Describe any assumptions made about any missing or unclear information.	Notes 1-2 Table 1
Study risk of bias assessment	11	Specify the methods used to assess risk of bias in the included studies, including details of the tool(s) used, how many reviewers assessed each study and whether they worked independently, and if applicable, details of automation tools used in the process.	Notes 1
Effect measures	12	Specify for each outcome the effect measure(s) (e.g. risk ratio, mean difference) used in the synthesis or presentation of results.	Notes 1
Synthesis methods	13a	Describe the processes used to decide which studies were eligible for each synthesis (e.g. tabulating the study intervention characteristics and comparing against the planned groups for each synthesis (item #5)).	Notes 1
	13b	Describe any methods required to prepare the data for presentation or synthesis, such as handling of missing summary statistics, or data conversions.	Notes 1, Sec Methods
	13c	Describe any methods used to tabulate or visually display results of individual studies and syntheses.	Notes 1, Sec Methods
	13d	Describe any methods used to synthesize results and provide a rationale for the choice(s). If meta-analysis was performed, describe the model(s), method(s) to identify the presence and extent of statistical heterogeneity, and software package(s) used.	Notes 1, Sec Methods
	13e	Describe any methods used to explore possible causes of heterogeneity among study results (e.g. subgroup analysis, meta-regression).	Notes 1,

2b) PRISMA 2020 Checklist

Section and Topic	Item #	Checklist item	Location where item is reported
			Sec Methods
	13f	Describe any sensitivity analyses conducted to assess robustness of the synthesized results.	Notes 3-4
Reporting bias assessment	14	Describe any methods used to assess risk of bias due to missing results in a synthesis (arising from reporting biases).	Notes 2
Certainty assessment	15	Describe any methods used to assess certainty (or confidence) in the body of evidence for an outcome.	Sec Results
**RESULTS**			
Study selection	16a	Describe the results of the search and selection process, from the number of records identified in the search to the number of studies included in the review, ideally using a flow diagram.	Fig 1a
	16b	Cite studies that might appear to meet the inclusion criteria, but which were excluded, and explain why they were excluded.	Data 1/1
Study characteristics	17	Cite each included study and present its characteristics.	Table 2
Risk of bias in studies	18	Present assessments of risk of bias for each included study.	Notes 2
Results of individual studies	19	For all outcomes, present, for each study: (a) summary statistics for each group (where appropriate) and (b) an effect estimate and its precision (e.g. confidence/credible interval), ideally using structured tables or plots.	Notes 1, Sec Methods
Results of syntheses	20a	For each synthesis, briefly summarise the characteristics and risk of bias among contributing studies.	Notes 2
	20b	Present results of all statistical syntheses conducted. If meta-analysis was done, present for each the summary estimate and its precision (e.g. confidence/credible interval) and measures of statistical heterogeneity. If comparing groups, describe the direction of the effect.	Notes 3, Fig. 1c, *meta-FSHD* app
	20c	Present results of all investigations of possible causes of heterogeneity among study results.	Notes 4
	20d	Present results of all sensitivity analyses conducted to assess the robustness of the synthesized results.	Notes 3-4
Reporting biases	21	Present assessments of risk of bias due to missing results (arising from reporting biases) for each synthesis assessed.	Notes 2
Certainty of evidence	22	Present assessments of certainty (or confidence) in the body of evidence for each outcome assessed.	Sec Results
**DISCUSSION**			
Discussion	23a	Provide a general interpretation of the results in the context of other evidence.	Sec Disc
	23b	Discuss any limitations of the evidence included in the review.	Sec Disc
	23c	Discuss any limitations of the review processes used.	Sec Disc
	23d	Discuss implications of the results for practice, policy, and future research.	Sec Disc
**OTHER INFORMATION**			
Registration and protocol	24a	Provide registration information for the review, including register name and registration number, or state that the review was not registered.	Sec Intro, Sec Methods

2b) PRISMA 2020 Checklist

Section and Topic	Item #	Checklist item	Location where item is reported
	24b	Indicate where the review protocol can be accessed, or state that a protocol was not prepared.	Sec Intro, Sec Methods
	24c	Describe and explain any amendments to information provided at registration or in the protocol.	Sec Intro, Sec Methods
Support	25	Describe sources of financial or non-financial support for the review, and the role of the funders or sponsors in the review.	Sec Ack
Competing interests	26	Declare any competing interests of review authors.	Sec CI
Availability of data, code and other materials	27	Report which of the following are publicly available and where they can be found: template data collection forms; data extracted from included studies; data used for all analyses; analytic code; any other materials used in the review.	Sec Methods

Reference:

Page, M. J. et al. The PRISMA 2020 statement: An updated guideline for reporting systematic reviews. The BMJ vol. 372. Available from: https://doi.org/10.1136/bmj.n71 (2021).

* Notes = Supplementary Notes
Tables = Supplementary Tables
Sec = Section
Intro = Introduction
Disc = Discussion
Ack = Acknowledgement
CI = Conflict of Interests

Supplementary Table 3: NMJ Associated Genes

Category (C)
S = Structure, Mantainance/ Formation
A = AChRs clustering
V = Vesicle transport
I = Ion channel

Upregulated	downregulated

Gene	C		Relation to the NMJ	References
SYT11	V	Synaptotagmin 11	(Next to *SYT3*) *SYT11* selectively recycles in dendrites. *SYT11* is a non-Ca^{2+} -binding *SYT*, which is proposed to function to ensure precision in vesicle retrieval, mainly by limiting the sites of membrane invagination at the early stage of endocytosis.	1, 2
TIMP2	S	TIMP Metallopeptidase Inhibitor 2	*TIMP2* regulates NMJ development via a beta1 integrin-mediated mechanism.	3
THBS4	S	Thrombospondin 4	It is reported, that *THBS4* is expressed in NMJs and its expression increases after muscle denervation. It is reported to regulate collagen expression and to participate in the formation and remodeling of the ECM.	4
TUBA1A	S	Tubulin Alpha 1a	Reduced *TUBA1A* was found to reduce NMJ synapses over time ultimately resulting in an adult-onset movement disorder in mice.	5
TNC	S	Tenascin C	Tenascin was found in the NMJs of the extrafusal fibres. Because of the close spatial relationship between tenascin and both sensory and motor nerve endings it was suggested that this glycoprotein is of functional importance in adult nerve-muscle contacts in human skeletal muscle. However, other researchers assume that	6, 7

			tenascin-C is dispensable for major aspects of synaptic development and regeneration.	
MAP1B	S	Microtubule Associated Protein 1B	*MACF1* anchors *MAP1b* and *EB1* at the postsynaptic membrane at neuromuscular synapses.	8
AGRN	S	Agrin	The proteoglycan Agrin activates *MuSK* (for Muscle-Specific Kinase), a receptor tyrosine kinase required for the formation and maintenance of the NMJ.	9, 10
KCNC4	I	Potassium Voltage-Gated Channel Subfamily C Member 4	Ion channel	11
LMNA	S	Lamin A/C	Lamin A/C, a scaffolding component of the nuclear envelope, was reported to be critical to maintaining the NMJ in mice. Nuclear dysfunction or deficiency was reported to lead to progressive NMJ degeneration in vivo contributing to NMJ deficits in aged muscles.	12
FGF18	A	Fibroblast Growth Factor 18	Secreted molecules like *Fgf18* (i.a.), which have been identified in the Wnt/β-catenin and fibroblast growth factors (FGF) signaling pathways, facilitate the clustering of AChRs by enhancing the Agrin-LRP4-MuSK signaling pathway. Lack of *FGF18* was reported to cause abnormal clustering of motor nerve terminals at the NMJ with reduced acetylcholine receptor clusters.	13, 14
ATP1A1	I	ATPase Na+/K+ Transporting Subunit Alpha 1	Na+/K+ -ATPase is an integral membrane protein responsible for establishing and maintaining the electrochemical gradients of Na and K ions across the plasma membrane. These gradients are essential i.a. for electrical excitability of nerve and muscle. Skeletal muscle contains primarily α2-subunits. Heterodimers that comprise different α – and β -subunits have distinct kinetic characteristics.	15
LIF	S	LIF Interleukin 6 Family Cytokine	*LIF* positively promotes the formation of NMJ in specific concentration range.	16

SPTLC2	S	Serine Palmitoyltransferase Long Chain Base Subunit 2	Sphingolipids were reported to regulate neuromuscular synapse structure and function in Drosophila. Mutations in the *SPT* enzyme subunit lace/*SPTLC2* have been shown to lead to deficits in synaptic structure and function.	17
HIF1A	S	Hypoxia Inducible Factor 1 Subunit Alpha	Hypoxia was shown to stimulate motor neuron regeneration and to accelerate NMJ re-innervation.	18
ATP1B3	I	ATPase Na+/K+ Transporting Subunit Beta 3	The β -subunit exists in three isoforms (β1–3). Skeletal muscle contains mainly β1- and β2-subunits, but the β3-subunit has also been detected. Heterodimers that comprise different α – and β -subunits have distinct kinetic characteristics.	15
GAS7	S	Growth Arrest Specific 7	*Gas7* was reported to be possibly involved in motor neuron function associated with muscle strength maintenance.	19
ERBB3	S	Erb-B2 Receptor Tyrosine Kinase 3	It has been shown that muscle neuregulin, together with *erbBs* (*erbB2* and *erbB3*; not *erbB4*) is aggregated in the postsynaptic apparatus and in the agrin-induced postsynaptic apparatus.	20
VAMP1	V	Vesicle Associated Membrane Protein 1	*VAMP1*, a member of the synaptobrevin family (a component of the SNARE complex) is involved in docking and fusion of the synaptic vesicles with presynaptic membrane in the NMJ.	21
MACF1	S	Microtubule actin cross linking factor 1	*MACF1*, which has binding sites for microtubules (MT) and actin, was demonstrated to be concentrated at the postsynaptic membrane at neuromuscular synapses, where it serves as a synaptic scaffold and organizing center for MT-associated proteins.	8
FHL1	A	Four And A Half LIM Domains 1	*FHL1* was reported to have a systemic effect on neuromuscular junction/ systemic regulation of AChR clustering and myotube formation via insulin-like growth factor and myostatin signaling pathways.	22

SQSTM1	S	Sequestosome 1	Fasting-induced skeletal muscle death, which has been shown to be accompanied by NMJ remodeling by increasing muscle-type *CHRN* turnover in a *TRIM63*-dependent manner, has been shown to imply increased production of endo-/lysosomal *CHRN* carriers that also contain the selective autophagy receptor *SQSTM1* (i.a.).	23
CADM3	S	Cell Adhesion Molecule 3	Synaptic adhesion molecules in *Cadm* family are reported to play a role at the NMJ.	24
LAMA5-AS1	S	Laminin α5 Antisense RNA1	Laminin forms laminin α4 and -α5 are typical of the synaptic BL.	25
SYT1	V	Synaptotagmin 1	Only *SYT* isoforms *SYT1* and *2* are targeted to synaptic vesicles. *SYT1* is reported to be the major Ca^{2+} sensor that triggers fast, synchronous neurotransmitter release upon Ca^{2+} binding.	1, 26
KCNE4	I	Potassium Voltage-Gated Channel Subfamily E Regulatory Subunit 4	Ion channel	11
PICALM	V	Phosphatidylinositol Binding Clathrin Assembly Protein	*PICALM* is involved in AP2-dependent clathrin-mediated endocytosis at the NMJ.	27
NCAM1	S	Neural Cell Adhesion Molecule 1	Structural alterations of NMJs have been reported in *NCAM*-deficient as well as transgenic mice expressing a GPI-anchored *NCAM* isoform that is normally found in developing and denervated muscle.	28, 29
PRICKLE2	S	Prickle Planar Cell Polarity Protein 2	Early deletion of *PRICKLE2* was reported to (i.a.) impair firing of action potentials.	30

KCNMB1	I	Potassium Calcium-Activated Channel Subfamily M Regulatory Beta Subunit 1	Ion channel	11
KCNAB1	I	Potassium Voltage-Gated Channel Subfamily A Regulatory Beta Subunit 1	Ion channel	11
ATP2A3	I	ATPase Sarcoplasmic/Endoplasmic Reticulum Ca2+ Transporting 3	It was documented that at least one member of the *SERCA3* family, h3d, is equally expressed in all the tissues examined, including cardiac and skeletal muscles, abolishing the idea of the non-muscle cell-specific expression of the *SERCA3* gene products. It is reported to transport calcium ions from the cytosol into the sarcoplasmic/endoplasmic reticulum lumen. Contributes to calcium sequestration within muscular excitation/contraction.	31
KCNN4	I	Potassium intermediate/small conductance calcium-activated channel, subfamily N, member 4	Ion channel	11
KCND1	I	Potassium Voltage-Gated Channel Subfamily D Member 1	Ion channel	11
DLG3	S	Discs Large MAGUK Scaffold Protein 3	Presynaptic *DLG* was shown to regulate synaptic function through the localization of voltage-activated Ca^{2+} Channels at the Drosophila larval NMJ.	32
CHRND	A	Cholinergic Receptor Nicotinic Delta Subunit	*CHRND* was shown to play a role in AChR clustering.	33

DOK7	A	Docking Protein 7	Upon activation by its ligand *agrin*, *MuSK* signals via the proteins casein kinase 2 (CK2), Dok-7 and Rapsyn to cluster acetylcholine receptors (AChR).	34
DNM2	S	Dynamin 2	Mutations in *DNM2* are reported to be a common cause of Centronuclear Myopathy. *DNM2*-mutation-containing zebrafish larvae were shown to have extensive triad abnormalities with abnormal distribution of T-tubules and terminal sarcoplasmic reticulum.	35
ANKRD23	S	Ankyrin Repeat Domain 23	It was shown that Nav1.4 Na+ channels are clustered at the NMJ through ankyrin scaffolding proteins and that the ankyrin-dependent Na+ channel clustering prevents neuromuscular synapse fatigue in mice. *ANKRD23* was reported to negatively regulate myoblast differentiation.	36, 37
SYN2	V	Synapsin II	Synapsin II is reported to have a specific role in preventing synaptic depression and maintaining the reserve pool of synaptic vesicles at central excitatory synapses and NMJ.	38
PPP1R12B	S	Protein Phosphatase 1 Regulatory Subunit 12B	It is also called Myosin Phosphatase-Targeting Subunit 2; In striated muscle phosphorylation of the regulatory light chain of myosin II (MLC) does not induce contraction but appears to play a modulatory role. The current hypothesis is that MLC phosphorylation in striated muscle increases Ca^{2+}-sensitivity, i.e. by inducing a shift to lower $[Ca^{2+}]_i$ in the force–Ca^{2+} relationship.	39
WNT4	A	Wnt Family Member 4	Analysis of the mouse *WNT4* -/- NMJ phenotype revealed severe innervation defects, with 30% of AChR clusters not enclosed by nerve terminals. It was also shown that loss of *WNT4* function resulted in a 35% decrease in the number of AChR clusters, whereas overexpression of *WNT4* in cultured myotubes increased the number of AChR clusters. In contrast, muscle structure and localization of several synaptic proteins, such as acetylcholinesterase, MuSK, and Rapsyn, were not disrupted in the *WNT4* mutant. However, MuSK was identified as a WNT4	40

			receptor, and WNT4 was shown not only to interact with the ectodomain of MuSK but also to mediate MuSK activation.	
COL4A5	S	Collagen Type IV Alpha 5 Chain	It has been described to form, together with *COL4A3* and *COL4A4*, triple helical α3:α4:α5 molecules, present at the synaptic BL of the NMJ.	41, 25
TRIM63	S	Tripartite Motif Containing 63	Fasting-induced skeletal muscle death has been shown to be accompanied by NMJ remodeling by increasing muscle-type *CHRN* turnover in a TRIM63-dependent manner.	23
KCNJ11	I	Potassium Inwardly Rectifying Channel Subfamily J Member 11	Ion channel	11
KCNC1	I	Potassium Voltage-Gated Channel Subfamily C Member 1	Ion channel	11
KCNK9	I	Potassium Two Pore Domain Channel Subfamily K Member 9	Ion channel	11
ATP1B1	I	ATPase Na+/K+ Transporting Subunit Beta 1	The β -subunit exists in three isoforms (β 1–3). Skeletal muscle contains mainly β1- and β2-subunits, but the β3-subunit has also been detected. Although the β -subunit does not possess catalytic activity, it is essential for assembly, maturation, and function of α/β -heterodimers.	15
SGCA	S	Sarcoglycan Alpha	SGCA is part of the multi-protein Dystrophin Glycoprotein Complex (DGC) that spans the sarcolemma and links cytoskeletal actin to the extracellular matrix. The complex is critical in maintaining the structural integrity of muscle fibers and the stability of the neuromuscular synapse.	42

KCNJ3	I	Potassium Inwardly Rectifying Channel Subfamily J Member 3	Ion channel	11
DVL1	A	Dishevelled Segment Polarity Protein 1	DVL1 and PAK1 form a ternary complex with *MuSK* which is important for *MuSK*-dependent regulation of AChR clustering during the formation of the NMJ.	43
DTNBP1	S	Dystrobrevin Binding Protein 1	*DTNBP1* was reported to have a role in neuromuscular synapse formation and maintenance.	44
GDNF	S	Glial Cell Derived Neurotrophic Factor	*GDNF* overexpression in muscle is reported to cause hyperinnervation of NMJs.	45
SYT13	V	Synaptotagmin 13	*SYT13* was shown to be a neuroprotective across motor neuron diseases like amyotrophic lateral sclerosis (ALS) and spinal muscular atrophy (SMA). *SYT13* gene therapy prolonged the lives of transgenic ALS mice by 14% and of SMA mice by 50%. Due to the complexity of the pathogenesis of motor neuron disease, it is believed to be beneficial in the context of combination therapy.	46, 47
SCN1B	I	Sodium Voltage-Gated Channel Beta Subunit 1	*SCN1B* contributes to the generation and propagation of action potentials in muscle and nerve cells. The heteromeric proteins consist of one alpha and two beta subunits, with the alpha subunit providing channel activity and the beta 1 subunit modulating the kinetics of channel inactivation.	48
CACNA1S	I	Calcium Voltage-Gated Channel Subunit Alpha1 S	Pore-forming alpha-1S subunit of the voltage-gated calcium channel that triggers L-type calcium currents in skeletal muscle. Calcium channels containing the alpha-1S subunit contribute important roles in coupling excitation and contraction in skeletal muscle via their interaction with RYR1, triggering the release of Ca^{2+} from the sarcoplasmic reticulum, which finally leads to muscle contraction.	49

CAPN3	S	Calpain 3	CAPN3, which is located in the sarcomere, is reported to be the only muscle-specific calpain that has important roles in the promotion of calcium release from skeletal muscle fibers, calcium uptake of sarcoplasmic reticulum, muscle formation and muscle remodelling. Recessive mutations in *CAPN3* are associated with limb-girdle muscular dystrophy type 2A.	50
RIC3	A	RIC3 Acetylcholine Receptor Chaperone	RIC3 is a transmembrane protein which acts as a molecular chaperone of nicotinic acetylcholine receptors (nAChRs).	51
CLCN1	I	Chloride Voltage-Gated Channel 1	In both autosomal dominant myotonia congenita and recessive generalized myotonia, mutations in *CLCN1*, which is reported to encode the chloride channel on skeletal muscle membrane, result in decreased sarcolemmal Cl^- conductance. This abnormal membrane property was reported to lead to myofiber hyperexcitability, repetitive firing of muscle action potentials, and clinical myotonia. Plays an important role in membrane repolarization in skeletal muscle cells after muscle contraction.	52, 53
SPEG	S	Striated Muscle Enriched Protein Kinase	SPEG, which is associated with a subgroup of Centronuclear Myopathy patients, was shown to be responsible for disruption of the excitation–contraction coupling (ECC) machinery, dysregulation of calcium homeostasis during ECC and impairment of muscle performance.	54
KCNT1	I	Potassium Sodium-Activated Channel Subfamily T Member 1	Ion channel	11
COL4A4	S	Collagen Type IV Alpha 4 Chain	It has been described to form, together with *COL4A3* and *COL4A5*, triple helical α3:α4:α5 molecules, present at the synaptic BL of the NMJ.	41, 25
COL4A3	S	Collagen Type IV Alpha 3 Chain	It has been described to form, together with *COL4A4* and *COL4A5*, triple helical α3:α4:α5 molecules, present at the synaptic BL of the NMJ.	41, 25

References

1. C. Dean, F. M. Dunning, H. Liu, E. Bomba-Warczak, H. Martens, V. Bharat, S. Ahmed, Edwin R. Chapman, Axonal and dendritic synaptotagmin isoforms revealed by a pHluorin-syt functional screen. *Mol Biol Cell.* **23**, 1715–1727 (2012).

2. C. Wang, Y. Wang, M. Hu, Z. Chai, O. Wu, R. Huang, W. Han, C. Xi Zhang, Z. Zhou, Synaptotagmin-11 inhibits clathrin-mediated and bulk endocytosis. *EMBO Rep* **17**, 47–63 (2016).

3. G. Lluri, G. D. Langlois, B. McClellan, P. D. Soloway, D. M. Jaworski, Tissue inhibitor of metalloproteinase-2 (TIMP-2) regulates neuromuscular junction development via a β1 integrin-mediated mechanism. *J Neurobiol.* **66**, 1365–1377 (2006).

4. K. Mäemets-Allas, M. Klaas, C. G. Cárdenas-León, T. Arak, E. Kankuri, V. Jaks, Stimulation with THBS4 activates pathways that regulate proliferation, migration and inflammation in primary human keratinocytes. *Biochem Biophys Res Commun.* **642**, 97–106 (2023).

5. G. Buscaglia, K. R. Northington, J. K. Moore, E. A. Bates, Reduced TUBA1A tubulin causes defects in trafficking and impaired adult motor behaviour. *eNeuro* **7**, ENEURO.0045-20.2020 (2020).

6. F. Pedrosa-Domellöf, I. Virtanen, L.-E. Thornell, Tenascin is present in human muscle spindles and neuromuscular junctions. *Neurosci Lett.* **198**, 173–176 (1995).

7. L. M. Moscoso, H. Cremer, J. R. Sanes, Organization and Reorganization of Neuromuscular Junctions in Mice Lacking Neural Cell Adhesion Molecule, Tenascin-C, or Fibroblast Growth Factor-5. *J Neurosci.* **18**, 1465–1477 (1998).

8. J. Oury, Y. Liu, A. Töpf, S. Todorovic, E. Hoedt, V. Preethish-Kumar, T. A. Neubert, W. Lin, H. Lochmüller, S. J. Burden, MACF1 links rapsyn to microtubule- and actin-binding proteins to maintain neuromuscular synapses. *J Cell Biol.* **218**, 1686–1705 (2019).

9. D. J. Glass, D. C. Bowen, T. N. Stitt, C. Radziejewski, J. Bruno, T. E. Ryan, D. R. Gies, S. Shah, K. Mattsson, S. J. Burden, P. S. DiStefano, D. M. Valenzuela, T. M. DeChiara, G. D. Yancopoulos, Agrin Acts via a MuSK Receptor Complex. *Cell.* **85**, 513–523 (1996).

10. M. Gautam, P. G. Noakes, L. Moscoso, F. Rupp, R. H. Scheller, J. P. Merlie, J. R. Sanes, Defective Neuromuscular Synaptogenesis in Agrin-Deficient Mutant Mice. *Cell.* **85**, 525–535 (1996).

11. L. A. Tintignac, H.-R. Brenner, M. A. Rüegg, Mechanisms Regulating Neuromuscular Junction Development and Function and Causes of Muscle Wasting. *Physiol Rev.* **95**, 809 –852 (2015).

12. N. Gao, K. Zhao, Y. Cao, X. Ren, H. Jing, G. Xing, W.-C. Xiong, L. Mei, A Role of Lamin A/C in Preventing Neuromuscular Junction Decline in Mice. *J Neurosci.* **40**, 7203–7215 (2020).

13. B. Ohkawara, M. Ito, K. Ohno, Secreted signaling molecules at the neuromuscular junction in physiology and pathology. *Int J Mol Sci.* **22**, 1–16 (2021).

14. K. Ito, B. Ohkawara, H. Yagi, H. Nakashima, M. Tsushima, K. Ota, H. Konishi, A. Masuda, S. Imagama, H. Kiyama, N. Ishiguro, K. Ohno, Lack of Fgf18 causes abnormal clustering of motor nerve terminals at the neuromuscular junction with reduced acetylcholine receptor clusters. *Sci Rep.* **8**, (2018).

15. S. Pirkmajer, A. V. Chibalin, ATPase regulation in skeletal muscle. *Am J Physiol Endocrinol Metab.* **311**, E1-E31 (2016).

16. T. Mars, Effects of LIF on Neuromuscular Junction Formation in Co-cultures of Rat Spinal Cord Explant and Human Muscle. *Croatica Chemica Acta.* **81**, 177–182 (2008).

17. R. J. H. West, L. Briggs, M. Perona Fjeldstad, R. R. Ribchester, S. T. Sweeney, Sphingolipids regulate neuromuscular synapse structure and function in Drosophila. *J Comp Neurol.* **526**, 1995–2009 (2018).

18. Y. Cho, J. E. Shin, E. E. Ewan, Y. M. Oh, W. Pita-Thomas, V. Cavalli, Activating Injury-Responsive Genes with Hypoxia Enhances Axon Regeneration through Neuronal HIF-1α. *Neuron.* **88**, 720–734 (2015).

19. B. T. Huang, P. Y. Chang, C. H. Su, C. C. K. Chao, S. Lin-Chao, Gas7-deficient mouse reveals roles in motor function and muscle fiber composition during aging. *PLoS One* **7**, (2012).

20. M. Rimer, I. Cohen, T. Lømo, S. J. Burden, U. J. McMahan, Neuregulins and erbB Receptors at Neuromuscular Junctions and at Agrin-Induced Postsynaptic-like Apparatus in Skeletal Muscle. *Mol Cell Neurosci.***12**, 1–15 (1998).

21. K. Polavarapu, S. Vengalil, V. Preethish-Kumar, G. Arunachal, S. Nashi, D. Mohan, T. Chawla, M. Bardhan, B. Nandeesh, P. Gupta, V. K. Gowda, H. Lochmüller, A. Nalini, Recessive VAMP1 mutations associated with severe congenital myasthenic syndromes – A recognizable clinical phenotype. *Eur J Paediatr Neurol.* **31**, 54–60 (2021).

22. J. Wu, K. Zhao, Z. Du, Y. Chen, F. Zhang, W. Jiang, J. Zheng, X. Wu, C. Shen, X. Xiao, Systemic effect of FHL1 on neuromuscular junction and myotube formation via insulin-like growth factor and myostatin signaling pathways. *Biochem Biophys Res Commun.* **537**, 125–131 (2021).

23. M. M. Khan, S. Strack, F. Wild, A. Hanashima, A. Gasch, K. Brohm, M. Reischl, S. Carnio, D. Labeit, M. Sandri, S. Labeit, R. Rudolf, Role of autophagy, SQSTM1, SH3GLB1, and TRIM63 in the turnover of nicotinic acetylcholine receptors. *Autophagy.* **10**, 123–136 (2014).

24. Y. Tanabe, E. Fujita, Y. K. Hayashi, X. Zhu, H. Lubbert, Y. Mezaki, H. Senoo, T. Momoi, Synaptic adhesion molecules in Cadm family at the neuromuscular junction. *Cell Biol Int.* **37**, 731–736 (2013).

25. B. L. Patton, Basal lamina and the organization of neuromuscular synapses. *J Neurocytol.* **32**, 883–903 (2003).

26. M. C. Shields, M. R. Bowers, H. L. Kramer, M. M. Fulcer, L. C. Perinet, M. J. Metz, N. E. Reist, The role of the C2A domain of synaptotagmin 1 in asynchronous neurotransmitter release. *PLoS One.* **15**, e0232991 (2020).

27. National Library of Medicine, “PICALM phosphatidylinositol binding clathrin assembly protein [Homo sapiens (human)]” (NIH, 2023; https://www.ncbi.nlm.nih.gov/gene/8301).

28. V. F. Rafuse, L. Polo-Parada, L. T. Landmesser, Structural and Functional Alterations of Neuromuscular Junctions in NCAM-Deficient Mice. *Journal of Neuroscience.* **20**, 6529–6539 (2000).

29. F. S. Walsh, C. Hobbs, D. J. Wells, C. R. Slater, S. Fazeli, Ectopic Expression of NCAM in Skeletal Muscle of Transgenic Mice Results in Terminal Sprouting at the Neuromuscular Junction and Altered Structure But Not Function. *Molecular and Cellular Neuroscience.* **15**, 244–261 (2000).

30. A. Dorrego-Rivas, J. Ezan, M. M. Moreau, S. Poirault-Chassac, N. Aubailly, J. De Neve, C. Blanchard, F. Castets, A. Fréal, A. Battefeld, N. Sans, M. Montcouquiol, The core PCP protein Prickle2 regulates axon number and AIS maturation by binding to AnkG and modulating microtubule bundling. *Sci. Adv.* **8**, 6333 (2022).

31. V. Martin, R. Bredoux, E. Corvazier, R. Van Gorp, T. Kovacs, P. Gelebart, J. Enouf, Three novel sarco/endoplasmic reticulum Ca 2+-ATPase (SERCA) 3 isoforms: Expression, regulation, and function of the members of the SERCA3 family. *Journal of Biological Chemistry.* **277**, 24442–24452 (2002).

32. C. Astorga, R. A. Jorquera, M. Ramírez, A. Kohler, E. López, R. Delgado, A. Córdova, P. Olguín, J. Sierralta, Presynaptic DLG regulates synaptic function through the localization of voltage-activated Ca2+ Channels. *Sci Rep* **6**, 32132 (2016).

33. J. S. Müller, S. K. Baumeister, U. Schara, J. Cossins, S. Krause, M. von der Hagen, A. Huebner, R. Webster, D. Beeson, H. Lochmüller, A. Abicht, CHRND mutation causes a congenital myasthenic syndrome by impairing co-clustering of the acetylcholine receptor with rapsyn. *Brain.* **129**, 2784–2793 (2006).

34. K. Okada, A. Inoue, M. Okada, Y. Murata, S. Kakuta, T. Jigami, S. Kubo, H. Shiraishi, K. Eguchi, M. Motomura, T. Akiyama, Y. Iwakura, O. Higuchi, Y. Yamanashi, The Muscle Protein Dok-7 Is Essential for Neuromuscular Synaptogenesis. *Science*. **312**, 1802–1805 (2006).

35. E. M. Gibbs, A. E. Davidson, W. R. Telfer, E. L. Feldman, J. J. Dowling, The myopathy-causing mutation DNM2-S619L leads to defective tubulation in vitro and in developing zebrafish. *Dis Model Mech*. **7**, 157. – 161 (2013).

36. C. Zhang, A. Joshi, Y. Liu, O. Sert, S. G. Haddix, L. H. Teliska, A. Rasband, G. G. Rodney, M. N. Rasband, Ankyrin-dependent Na+ channel clustering prevents neuromuscular synapse fatigue. *Current Biology*. **31**, 3810-3819.e4 (2021).

37. X. Wang, R. Zeng, H. Xu, Z. Xu, B. Zuo, The nuclear protein-coding gene ANKRD23 negatively regulates myoblast differentiation. *Gene*. **629**, 68–75 (2017).

38. L. Medrihan, F. Cesca, A. Raimondi, G. Lignani, P. Baldelli, F. Benfenati, Synapsin II desynchronizes neurotransmitter release at inhibitory synapses by interacting with presynaptic calcium channels. *Nat Commun*. **4**, 1512 (2013).

39. R. Okamoto, T. Kato, A. Mizoguchi, N. Takahashi, T. Nakakuki, H. Mizutani, N. Isaka, K. Imanaka-Yoshida, K. Kaibuchi, Z. Lu, K. Mabuchi, T. Tao, D. J. Hartshorne, T. Nakano, M. Ito, Characterization and function of MYPT2, a target subunit of myosin phosphatase in heart. *Cell Signal*. **18**, 1408–1416 (2006).

40. L. Strochlic, J. Falk, E. Goillot, S. Sigoillot, F. Bourgeois, P. Delers, J. Rouvière, A. Swain, V. Castellani, L. Schaeffer, C. Legay, Wnt4 participates in the formation of vertebrate neuromuscular junction. *PLoS One*. **7**, e29976 (2012).

41. M. Kjær, Role of Extracellular Matrix in Adaptation of Tendon and Skeletal Muscle to Mechanical Loading. *Physiol Rev*. **84**, 649–698 (2004).

42. D. C. Belhasan, M. Akaaboune, The role of the dystrophin glycoprotein complex on the neuromuscular system. *Neurosci Lett*. **722**, 134833 (2020).

43. UniProt Consortium, "O14640 · DVL1_HUMAN" (UniProt, 2023; https://www.uniprot.org/uniprotkb/O14640/entry#function).

44. J. X. Tang, J. Zhou, J. B. Fan, X. W. Li, Y. Y. Shi, N. F. Gu, G. Y. Feng, Y. L. Xing, J. G. Shi, L. He, Family-based association study of DTNBP1 in 6p22.3 and schizophrenia. *Mol Psychiatry*. **8**, 717–718 (2003).

45. Q. T. Nguyen, A. S. Parsadanian, W. D. Snider, J. W. Lichtman, Hyperinnervation of Neuromuscular Junctions Caused by GDNF Overexpression in Muscle. *Science*. **279**, 1725–1729 (1998).

46. M. Nizzardo, M. Taiana, F. Rizzo, J. Aguila Benitez, J. Nijssen, I. Allodi, V. Melzi, N. Bresolin, G. P. Comi, E. Hedlund, S. Corti, Synaptotagmin 13 is neuroprotective across motor neuron diseases. *Acta Neuropathol.* **139**, 837–853 (2020).

47. I. Fyfe, Synaptotagmin 13 — the key to cell resilience in motor neuron disease? *Nat Rev Neurol.* **16**, 186 (2020).

48. National Library of Medicine, “SCN1B sodium voltage-gated channel beta subunit 1 [Homo sapiens (human)]” (NIH, 2023; https://www.ncbi.nlm.nih.gov/gene?Cmd=DetailsSearch&Term=6324).

49. UniProt consortium, “Q13698 · CAC1S_HUMAN” (UniProt, 2023; https://www.uniprot.org/uniprotkb/Q13698/entry#function).

50. L. Chen, F. Tang, H. Gao, X. Zhang, X. Li, D. Xiao, CAPN3: A muscle-specific calpain with an important role in the pathogenesis of diseases (Review). *Int J Mol Med.* **48**, 203 (2021).

51. N. S. Millar, RIC-3: a nicotinic acetylcholine receptor chaperone. *Br J Pharmacol.* **153**, 177–183 (2008).

52. C. Fahlke, C. L. Beck, A. L. George, A mutation in autosomal dominant myotonia congenita affects pore properties of the muscle chloride channel. *Physiology*. **94**, 2729–2734 (1997).

53. G. Ulzi, M. Lecchi, V. Sansone, E. Redaelli, E. Corti, D. Saccomanno, S. Pagliarani, S. Corti, F. Magri, M. Raimondi, G. D'Angelo, A. Modoni, N. Bresolin, G. Meola, E. Wanke, G. P. Comi, S. Lucchiari, Myotonia congenita: Novel mutations in CLCN1 gene and functional characterizations in Italian patients. *J Neurol Sci.* **318**, 65–71 (2012).

54. K. G. Espinosa, S. Geissah, L. Groom, J. Volpatti, I. C. Scott, R. T. Dirksen, M. Zhao, J. J. Dowling, Characterization of a novel zebrafish model of SPEG-related centronuclear myopathy. *Dis Model Mech.* **15**, dmm049437 (2022).
