# Supplementary material for: Meta-analysis towards FSHD reveals misregulation of neuromuscular junction, nuclear envelope, and spliceosome
Source: Commun Biol. 2024 May 25;7:640. doi: 10.1038/s42003-024-06325-z (PMC11127974; doi:10.1038/s42003-024-06325-z)
Supplement: Supplementary file 3 — Desription of Additional Supplementary Files [file 42003_2024_6325_MOESM3_ESM.docx]

Description of Additional Supplementary Files

**File name:** Supplementary Data 1.xlsx

**Description:** Complete results of the analyses

- Sheet 1: Inclusion and exclusion of studies
- Sheet 2: Significant genes across all datasets
- Sheet 3: Random effects model enrichment analysis results
- Sheet 4: Vote counting approach
- Sheet 5: GO terms for upregulated pathways in the vote counting approach
- Sheet 6: GO terms for downregulated pathways in the vote counting approach
- Sheet 7: MAJIQ results
- Sheet 8: GSE115650 DESeq2 analysis
- Sheet 9: Nuclear Envelope Transmembrane Proteins (NETs)
